# Integrated spatial omics of metabolic reprogramming and the tumor microenvironment in pancreatic cancer

**DOI:** 10.1016/j.isci.2025.112681

**Published:** 2025-05-15

**Authors:** Hao Wu, Qiyao Zhang, Zhen Cao, Hongtao Cao, Mengwei Wu, Mengdi Fu, Tingping Huang, Xianlin Han, Xiaoyan Chang, Ziwen Liu

**Affiliations:** 1 Department of General Surgery, Peking Union Medical College Hospital, Chinese Academy of Medical Sciences and Peking Union Medical College, Beijing 100730, P.R. China; 2 Department of General Surgery, Beijing Hospital, National Center of Gerontology, Institute of Geriatric Medicine, Chinese Academy of Medical Sciences, Beijing 100730, P.R. China; 3 Department of Clinical Medicine, Peking Union Medical College Hospital, Chinese Academy of Medical Sciences and Peking Union Medical College, Beijing 100730, P.R. China; 4 Lung Transplantation Center, The Affiliated Wuxi People’s Hospital of Nanjing Medical University, Wuxi People’s Hospital, Wuxi Medical Center, Nanjing Medical University, Nanjing 214000, P.R. China; 5 Department of Pathology, Peking Union Medical College Hospital, Chinese Academy of Medical Sciences and Peking Union Medical College, Beijing 100730, P.R. China

**Keywords:** Cancer, Metabolomics, Microenvironment, Transcriptomics

## Abstract

Metabolic reprogramming is a defining feature of pancreatic cancer, influencing tumor progression and the tumor microenvironment. By integrating single-cell transcriptomics, spatial transcriptomics, and spatial metabolomics, this study visualized the spatial co-localization of metabolites and gene expression within tumor samples, uncovering metabolic heterogeneity and intercellular interactions. Spatial transcriptomics identified distinct pathological regions, which were further characterized using single-cell transcriptomic data and pathologist annotations. Pseudotime trajectory analysis revealed metabolic shifts along the malignant progression, while single-cell Metabolism (scMetabolism) delineated metabolic differences between pathological regions, classifying them as hypermetabolic or hypometabolic. Notably, aberrant cell communication between cancer cells, macrophages, and fibroblasts was observed, with key receptor-ligand pairs significantly co-expressed in malignant regions and correlated with poor prognosis. Spatial metabolomics imaging identified signature metabolites, highlighting metabolic alterations in amino acid metabolism, polyamine metabolism, fatty acid synthesis, and phospholipid metabolism. This integrated analysis provides critical insights into pancreatic cancer metabolism, offering potential avenues for targeted therapeutic interventions.

## Highlights

• Integrated spatial transcriptomic and metabolomic analysis of pancreatic cancer
• Multi-omics analysis of amino acid and lipid metabolism reveals metabolic heterogeneity
• Identified specific metabolic pathways and scored activity to distinguish metabolic regions
• Characterized ligand–receptor crosstalk in metabolic regions and assessed prognostic value

## Graphical abstract

**Figure:**
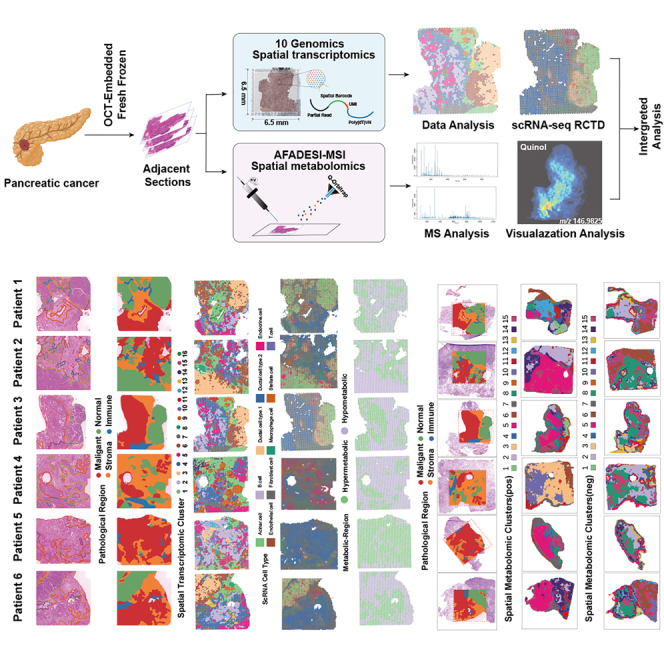

## Introduction

Pancreatic ductal adenocarcinoma (PDAC) is a highly malignant tumor with a poor prognosis. It has the characteristics of insidious onset, difficulty in early diagnosis, rapid progression, and frequent local invasion and distant metastasis.^1^ In recent years, the incidence and mortality of pancreatic cancer have continued to rise worldwide. The latest statistics from the American Cancer Society show that in 2024, it is expected that the number of new cases of pancreatic cancer in the United States will reach 66,400, and the number of deaths will exceed 60,000. The overall five-year survival rate is less than 9%, ranking fourth among patient deaths caused by malignant tumors. It is expected that by 2030, pancreatic cancer will rank second in malignant tumor-related deaths in the United States.^2^ There is an urgent need to develop accurate and effective comprehensive diagnosis and treatment strategies for pancreatic cancer.^3^

Metabolic reprogramming has gradually been recognized as one of the hallmarks of cancer. It refers to the adaptive changes in the balance of anabolism and catabolism that occur in tumor cells during malignant proliferation in order to meet the large demand for substances and energy. This allows tumor cells to gain a survival advantage in the nutrient-poor tumor microenvironment (TME) caused by rapid proliferation.^4^ Since the discovery of the Warburg effect by Otto Warburg in the early 20th century, the study of tumor metabolism has begun. The various metabolic reprogramming mechanisms of tumor cells have been gradually elucidated, and they can use non-traditional pathways to promote growth and proliferation and serve as raw materials for the *de novo* synthesis of nucleic acids, lipids, and proteins. People have gradually discovered that tumor metabolism is an overall change in the metabolic network at multiple levels, and the metabolic interactions between tumors and surrounding cells have a significant impact on cancer progression and anti-tumor immune response. In-depth research on metabolic disorders in the occurrence and development of pancreatic cancer, finding metabolic differences and key targets, clarifying the molecular mechanisms that regulate metabolic reprogramming in pancreatic cancer, and targeted intervention of key regulatory molecules in the metabolic reprogramming process are expected to become new diagnostic targets and therapeutic strategies.

However, due to the complexity of the cellular metabolic network, the heterogeneity of the TME, and the diversity of intercellular metabolic communication, it is still challenging to visualize tumor metabolic reprogramming and intercellular interactions from a spatially resolved multi-omics level. The development of new technologies in recent years has made this possible. Spatial transcriptomics (ST) can obtain transcriptome expression profiles at a resolution close to the single-cell level while retaining the spatial location information of each cell on the tissue section. At the same time, combining scRNA-seq data for annotation can further clarify and explore the relationship between various types of cells in the TME. In addition, the gradually developed spatial metabolomics (SM) based on airflow-assisted desorption electrospray ionization-mass spectrometry imaging (AFADESI-MSI) can identify small molecule metabolites in tissue sections at micron resolution with high throughput while retaining spatial location information. In this study, we adopted the above-mentioned comprehensive spatially resolved multi-omics approach to elucidate the main metabolic reprogramming that occurs in PDAC at the metabolic and transcriptional levels, and at the same time gain a deep understanding of the biochemical heterogeneity within the tumor and its key role in tumor growth and development.

## Results

### The ST profiles of PDAC reveal intratumor heterogeneity

To investigate the spatial multi-omics characteristics of pancreatic cancer tissue and reveal key molecular events in the process of malignant transformation, we collected fresh tissue samples from 6 patients who were clinically diagnosed with pancreatic cancer and underwent surgical resection (Table S1). Consecutive frozen slices were prepared according to the protocol and stained with H&E. Based on H&E staining, experienced pathologists selected representative areas of the samples containing malignant tumors, acinar or normal ductal epithelium, immune infiltration areas, and stromal tissue. According to the maximum slice area detected by the platform, two adjacent continuous slices of tissue were selected for SM analysis with a size of 10 × 10 mm and ST analysis was further performed on a representative area with a size of 6.5 × 6.5 mm.

**Figure 1:**
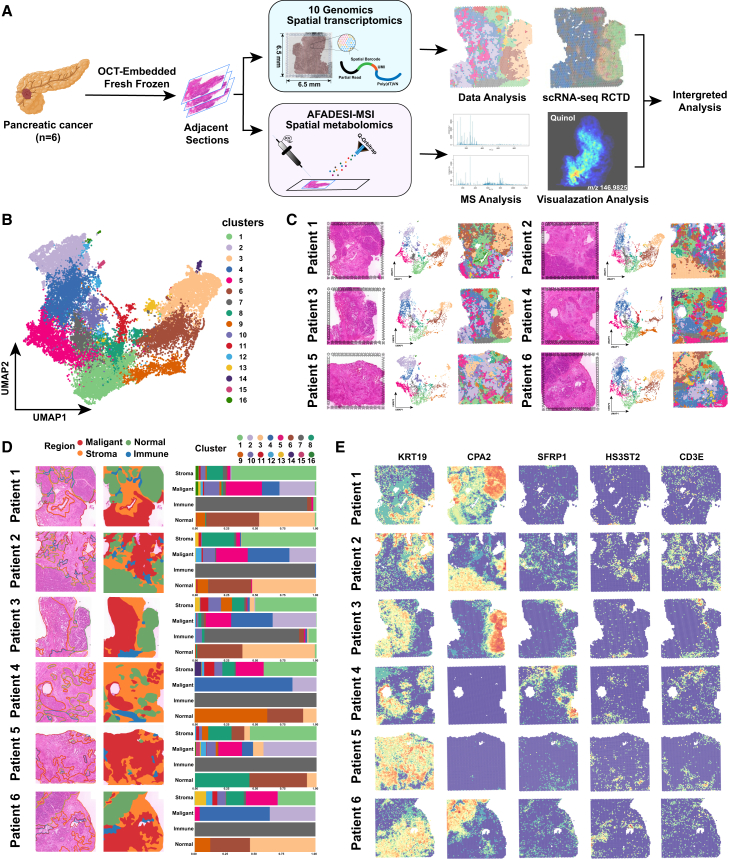
Flow chart and spatial transcriptomic landscape of pancreatic cancer (A) Schematic diagram exhibiting the detection and analysis of ST and SM in pancreatic cancer. (B) Unsupervised clustering analysis UMAP divided all spots from six samples into 16 clusters. (C) H&E staining images (left), UMAP plots (middle), and ST feature plots (right) of 16 clusters in six samples. (D) The tissue of six samples was divided into four regions based on the histopathological features, including malignant, normal, immune, and stroma regions. H&E staining images (left), ST images of spots with tissue regions annotated by different colors (middle), and the proportion of spots and corresponding clusters in each tissue region (right) were presented, respectively. (E) ST feature plots showing the expression of representative marker genes in each spot of different tissue regions.

The schematic diagram of the sample collection and spatial multi-omics integrated analysis process is shown in the figure (Figure 1A). By using the 10× Genomics Visium platform for ST (Figure S1A), a total of 25,928 spots were detected from 6 samples, with an average of 35,391 unique molecular identifiers (UMIs) per spot, representing 6,127 genes. After integrating and normalizing the spots of each sample, unsupervised clustering analysis was performed using uniform manifold approximation and projection (UMAP), and the results showed that there was no obvious batch effect between samples (Figure S1C). All spots in the 6 samples were divided into 16 clusters (Figure 1B), and different sub-regions were divided according to the spatial distribution of each spot on the tissue section and the tissue pathological structure (Figure S2). Among them, the malignant region is mainly located in the tumor region, including several clusters (C2, C4, C5, C10, and C11); the normal region is mainly located in the acinar and normal duct epithelial area, including some clusters (C3, C6, and C9); the stroma region is mainly located in the tumor stroma and connective tissue area, including some clusters (C1, C8); the immune region is mainly located in the immune infiltration area, mainly including cluster 7.

Next, we annotated the biological characteristics of each cluster based on the top 10 differentially expressed marker genes, revealing transcriptional similarities between different subtypes (Figure S1D). Specifically, in cluster 1, we found that the chemokine CCL19 produced by fibroblasts and markers of tumor-associated fibroblasts such as SFRP1, PI16, and ADH1B were significantly expressed. Correspondingly, in cluster 8, we can find that SGCA is significantly expressed, which is considered to be a marker of pancreatic stellate cells (PSCs). In cluster 3, we can observe the upregulation of pancreatic acinar-related genes FGL1 and AMY2A; and in cluster 6, in addition to FGA and FGG related to alpha cells, SERPINA6 related to ductal cells is also significantly upregulated. In cluster 7, multiple macrophage-related markers were upregulated, such as LY86, AQP9, and BCL2A1. In addition, we also show the markers of corresponding cell types in other clusters (Figure S3).

In summary, we identified 16 different clusters for PDAC tissues and divided them into four different regions including malignant region, normal region, stromal region, and immune region. By combining transcriptome data with spatial information and corresponding histopathological features, we laid the foundation for further analysis of cell type diversity, cell evolution, and intercellular communication.

### Spatio-temporal evolution model of PDAC

According to previous literature, there are different hypotheses about the progression of pancreatic cancer. We constructed a spatiotemporal evolution model based on the transcriptional profile during the progression from acinar to malignant ductal cells. Because the spatial resolution of ST is limited, we integrated the scRNA-seq data previously published by our center (PRJCA001063)^5^ and performed SPOTlight analysis on each spot (Figure S4A).

First, we re-annotated the cell types on the PRJCA001063 data and obtained 10 cell types, including acinar cell, ductal cell type 1, ductal cell type 2, endocrine cell, endothelial cell, fibroblast cell, macrophage cell, stellate cell, B cell, and T cell, as well as their characteristic gene expression features. Then, we deconvoluted the 10 cell types for each spot in the ST data and visualized the top 1 (Figure S4B) and top 2 (Figure S4C) cells in each spot. At the same time, we calculated the proportions of various cells in different spots corresponding to clusters (Figure S4D) and the interactions among different cell types (Figure S4E), reflecting the complex TME and intercellular communication of pancreatic cancer. In addition, we visualized the different cell types in the tissue sections of each patient (Figure S5).

**Figure 2:**
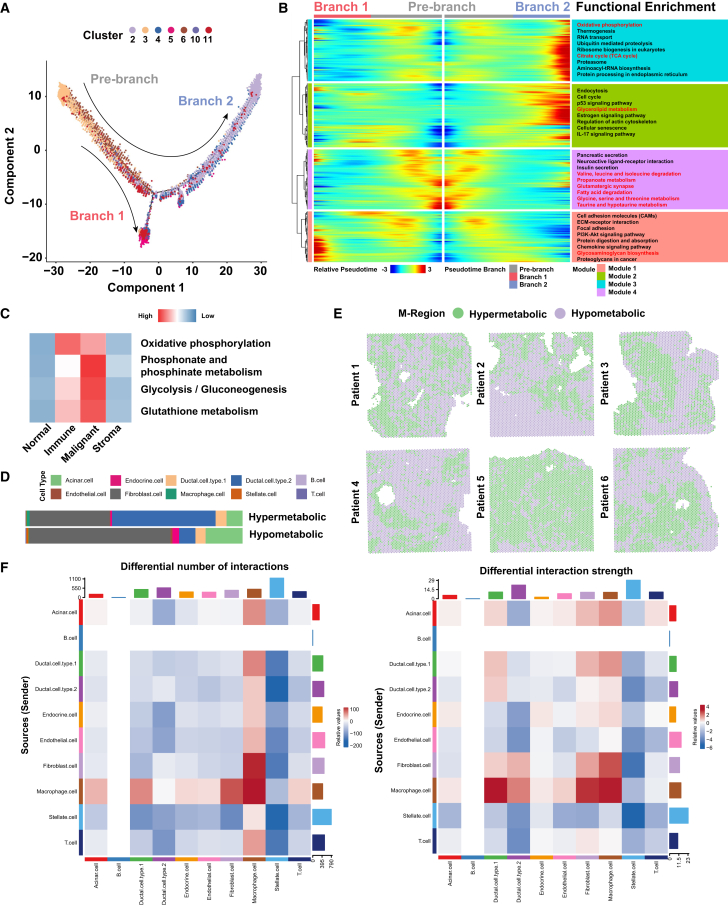
Pseudotime analysis, scMetabolism, and cell communication reveal metabolic reprogramming and interactions (A) Trajectory reconstruction of PDAC consisted of three branches: pre-branch (before bifurcation), T1 branch (bottom), and T2 branch (top). Each point corresponds to a spot. (B) BEAM heatmap plot displaying the expression patterns of pseudotime-specific genes and the corresponding GO pathway terms (hierarchically clustered into three profiles) in the malignant trajectory. (C) Heatmap shows the metabolic score representing glycolysis, pentose phosphate pathway, oxidative phosphorylation, and glutathione metabolism for each region. All spots were categorized into two clusters based on their metabolic activity: hypermetabolic, and hypometabolic. (D) Deconvolution results indicate the proportion of representative cell type of each spot in different types of metabolic regions. (E) Representative spatial transcriptomic tissue slides show the spatially projected metabolism clusters. (F) Heatmap showing the counts and strength of cell-cell communication between different cell types in the two metabolic regions.

To explore the spatiotemporal transcriptomic characteristics of pancreatic cancer malignant transformation, we selected a total of 17,398 spots (mainly clusters 2, 3, 4, 5, 6, 10, and 11) from malignant and normal regions (Figure S6) and used Monocle2 for pseudotime analysis, while other clusters were excluded from subsequent pseudotime analysis due to lack of epithelial cell distribution or small number of spots. We constructed the malignant progression trajectory of PDAC (Figure 2A), in which the initiation (front branch) of PDAC occurred in the acinar (from clusters 3 and 6). Cluster 2 and cluster 5 play a decisive role in influencing the evolutionary direction of branch 1 and branch 2.

To further explore the temporal and functional differences in the malignant trajectory of pancreatic cancer, we attempted to annotate the differentially expressed genes (DEGs) in the spots on the two branches. Pre-branch mainly shows biological functions of pancreatic secretion and insulin secretion. Enrichment of metabolism-related pathways (such as fatty acid degradation, taurine, and hypotaurine metabolism, and several amino acid metabolism pathways) can also be observed. In branch 2, tumor cells show significant enrichment of cell cycle, RNA transport, and translation-related genes. In terms of metabolism, module 2 can be enriched in glycerolipid metabolism, but module 3 is enriched in oxidative phosphorylation and TCA cycle, suggesting that metabolic heterogeneity seems to exist even in tumor cells. The branch is more like an acinar-ductal metaplasia (ADM) state, functionally combining the characteristics of both, with significant enrichment of glycosaminoglycan biosynthesis (Figure 2B).

### ST reveals metabolic heterogeneity and cellular interactions in PDAC

Based on the suggestion that pancreatic cancer progression may be accompanied by metabolic reprogramming, we evaluated the metabolic activity of each spot according to the pathological regions using scMetabolism and observed significant differences between the different partitions (Figure S7).

In addition, we performed functional variation analysis based on SM, calculated the scores of related pathways at each pixel, and visualized them. Subsequently, we selected four metabolic pathways (glycolysis/gluconeogenesis, pentose phosphate pathway, oxidative phosphorylation, and glutathione metabolism) based on the consistent trends and biological behavior of ST and SM (Figure S8). The metabolic scores of the four pathways were calculated by scMetabolism, and the average metabolic score was used as the metabolic feature, representing the metabolic activity of each spot in the tissue. All spots were clustered into two categories: hypermetabolism and hypometabolism (Figures 2C–2E). Cell types have different metabolic tendencies. For example, macrophages and ductal cell type 2 tend to accumulate in hypermetabolism regions, while fibroblasts do the opposite. We found that even in the malignant region, there was a relatively high and low metabolic activity, and we further explored the communication in different metabolic regions and the mechanism of metabolic subtypes of PDAC.

**Figure 3:**
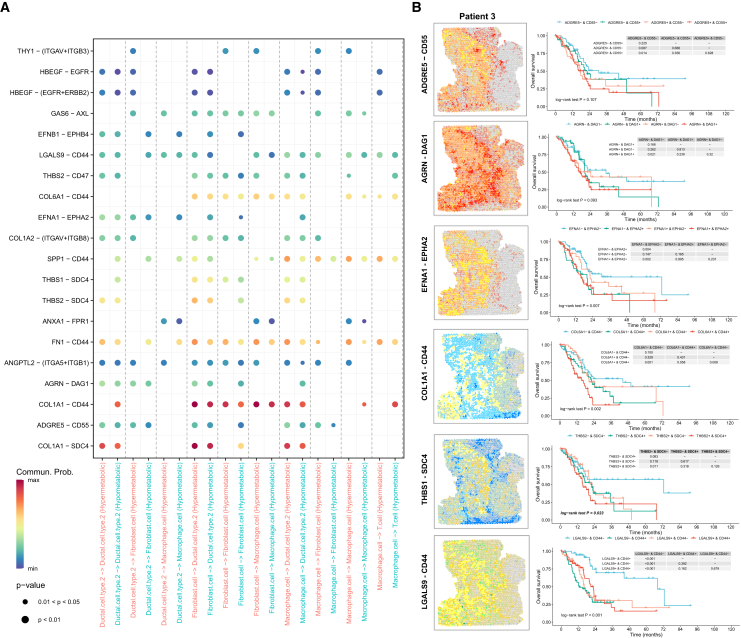
Interactions between cell types in different metabolic regions and potential clinical significance (A) Dot plots showing ligand–receptor pairs that are significantly expressed in hypermetabolic regions. (B) ST feature plots exhibiting the expression level and spatial distribution of representative ligand–receptor pairs. The correlation between transcriptomic level and prognosis were also presented.

Communication between different cells in TME is the main mechanism of interaction, so we analyzed the cell-cell interactions in different metabolic regions. We found that the communication intensity between macrophages, fibroblasts, and ductal cell type 2 in the hypermetabolism region was significantly higher than that in the hypometabolism region. Then we used the ligand-receptor analysis algorithm CellChat to perform ligand-receptor interaction analysis on the above cells. We screened out the ligand-receptor pairs with significant interactions in the hypermetabolism region (Figure 3A). The expression pattern of representative ligand-receptor pairs was displayed by ST (Figure 3B). We performed a clinical relevance analysis on the representative ligand-receptor pairs in the PAAD data from TCGA and found that the ligand-receptor pairs represented by ADGRE5-CD55, AGRN-DAG1, EFNA1-EPHA2, COL1A1-CD44, THBS1-SDC4, and LGALS9-CD44 were expressed at a higher level in tumor tissues compared with normal tissues, and the high expression of ligand-receptor pairs was associated with poor prognosis (Figures 3B and S9–S11).

In addition, we also performed SECNIC analysis on duct cell type 2 cells in hypermetabolic and hypometabolic regions to explore potential driving factors (Figure S12). There are notable differences in the regulon activity scores (RAS) between regions. In the hypermetabolic regions, regulators such as SREBF2, SREBF1, and NHF4A show increased activity. In contrast, the hypometabolic regions exhibits higher activity scores for RBPJL, IRF3, and NR5A2 (Figures S12A and S12B). In the regulon specificity score (RSS) analysis, CEBPA and PTF1A regulators appear to be specifically associated with the hypermetabolic and hypometabolic regions, respectively (Figure S12C). Subsequently, association clustering was performed, and the connection specificity index (CSI) was used to assess the relationships between different regulons. Modules 1 and 3 exhibit similar gene expression patterns, while modules 2 and 4 show a trend of differentiation (Figures S12D and S12E).

### SM atlas reveals metabolic reprogramming in PDAC

**Figure 4:**
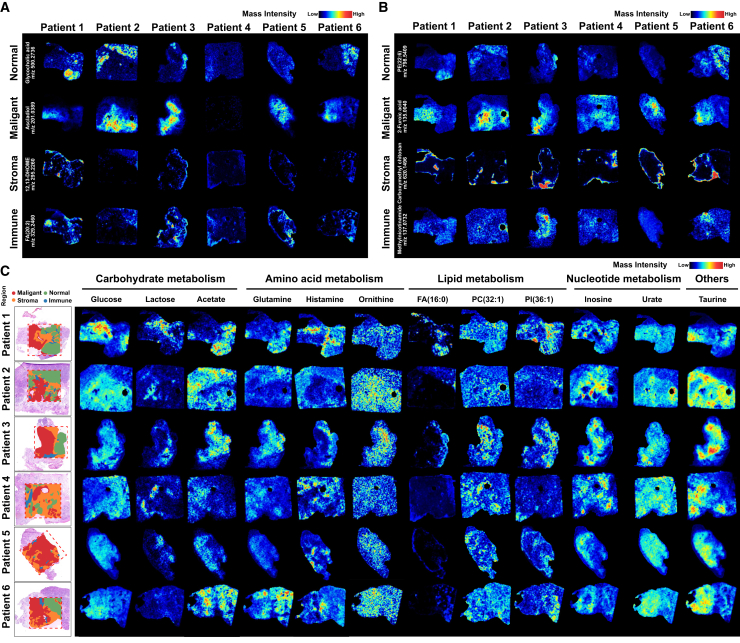
Spatial metabolomic atlas of pancreatic cancer (A and B) MSI images showing the abundance and distribution of representative metabolites and m/z information in each histopathological region in negative pattern (A) and positive pattern (B). (C) MSI images exhibiting the abundance of representative differential metabolites in different regions.

We established the SM landscape of PDAC using the AFADESI-MSI platform (Figure 4). We annotated the m/z information with metabolites, annotating 522 and 642 metabolites in the cation and anion modes, respectively (Table S2). Spatial shrunken centroids clustering (SSCC) was performed on the quantitative matrix to visualize the clustering results of the overall expression levels of differential metabolites in different tissue regions. All detected variables were decomposed into 15 clusters to reveal the main spatial characteristics of tissue metabolites, indicating that there is significant intratumor metabolomic heterogeneity in pancreatic cancer tissues (Figures S13A–S13G). According to the histopathological structure of HE staining and combined with the SSCC clustering results, the target area was selected for subsequent analysis.

By using orthogonal partial least squares discriminant analysis (OPLS-DA), we identified differentially enriched metabolites (DEMs) within each region, and some metabolites were significantly different in the malignant region compared to other regions. We then displayed the top 50 most significantly different m/z within each region (Figure S14). We visualize some of these representative metabolites.

The Warburg effect is one of the most typical metabolic changes in tumor cells. Through the presentation of carbohydrate metabolism (Figure S15), we found that the content of glucose (m/z 215.0329) in the malignant region increased significantly, and lactic acid (m/z 89.0242) was also significantly enriched, both of which reflected the characteristics of rapid glycolysis metabolism in tumor cells. However, at the same time, some metabolites such as acetic acid (m/z 96.9702) and malic acid (m/z 168.9903) also increased in the malignant region, but there was metabolic heterogeneity.

Amino acid metabolism plays an important role in pancreatic tissue metabolism (Figures S16 and S17). Due to the functional characteristics of pancreatic acinar cells, some amino acids tend to be enriched in normal regions, such as glycine (m/z 74.0249), alanine (m/z 88.0401), proline (m/z 114.0570), and arginine (m/z 347.21552). Glutamine (m/z 145.0615) is the main source of carbon and nitrogen for cancer cells, which can be converted to glutamate (m/z 146.0431) in mitochondria. It is upregulated in the malignant region compared to other amino acids, but still lower than the normal region, and there is heterogeneity between individuals and within tissues. Aggressive tumor tissues are characterized by higher glutamine consumption, so the availability of glutamine in tissues is low.^6^ In addition, amino acid metabolites such as ornithine (m/z 131.0831) were significantly accumulated in the malignant region, suggesting a possible active polyamine metabolic signature. In the stroma and immune regions, enriched histidine (m/z 112.0876) was observed, which is believed to be related to the immunosuppressive microenvironment.

Lipid metabolism can meet the high energy demand of tumor cell proliferation (Figures S18–S22). Lipid metabolism can meet the high energy demand of tumor cell proliferation. Free fatty acids represented by palmitic acid (FA 16:0, m/z 255.2321) and stearic acid (FA 18:0, m/z 305.2477) are significantly downregulated in malignant regions, which may regulate cell apoptosis.^7^ Phospholipids are important components of the cell membrane system, and there is usually significant phospholipid metabolic reprogramming during tumor progression. PC (32: 1) (m/z 754.5372) and others are enriched in the malignant region, while PG (38: 0) (m/z 851.5768) and LysoPC (16: 0) (m/z 518.3215) etc. are enriched in the normal region. Interestingly, PC (35: 4) (m/z 766.5343), PI (36: 1) (m/z 885.5453), and PS (38: 3) (m/z 794.5369) etc. are enriched in the stroma/immune region.

In terms of nucleotide metabolism (Figure S23), urate (m/z 167.0215) and inosine (m/z 267.0747) were significantly upregulated in all malignant regions, while adenine (m/z 134.0466) was relatively reduced. We also visualized some other metabolites (Figure S24), such as taurine (m/z 124.0069) was significantly enriched in malignant regions, while ascorbic acid (m/z 175.0238) was the opposite.

### Integrative analysis of amino acid metabolism disorders

**Figure 5:**
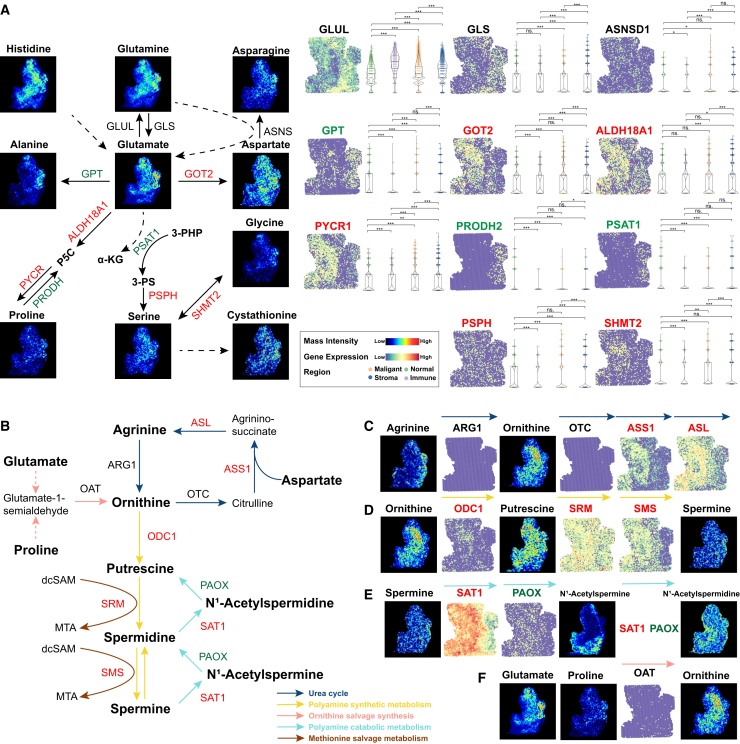
Visualization of metabolic reprogramming of amino acid metabolism in pancreatic cancer (A) The interconnected pathways of amino acids (AAs) metabolism. Glutamine and glutamate have a central role and can each be used for the synthesis of other AAs. Glutamate can be utilized to generate alanine, aspartate, serine, proline and also histidine. Aspartate is further utilized to generate asparagine. Serine makes glycine and donates methyl groups for one-carbon metabolism. Serine can also generate cysteine via the *trans*-sulfuration pathway. Violin plot show expression levels of key genes in amino acid metabolism. (B) Schematic maps of polyamine metabolism, including urea cycle (blue arrows), polyamine synthetic metabolism (yellow arrows), ornithine salvage synthesis (red arrows), polyamine catabolic metabolism (green arrows), and methionine salvage metabolism (brown arrows). (C–F) The spatial distribution feature of metabolic products and enzymes in the urea cycle (C), polyamine synthetic metabolism (D), polyamine catabolic metabolism (E) and ornithine salvage synthesis (F). Symbols: ns. denotes non-statistically significant, ∗ indicates a *p*-value <0.05, ∗∗ represents a *p*-value <0.01, and ∗∗∗ signifies a *p*-value <0.001. Data are represented as mean ± SEM.

Amino acid metabolism can affect cancer cell status and systemic metabolism through energy metabolism and signal transduction. The synthesis or consumption of amino acids is the result of a complex interaction between tissue-specific gene expression programs, dietary consumption, and local consumption/secretion rates. This results in the intrinsic complexity of amino acid metabolism. Glutamine is the amino acid with the highest consumption rate in PDAC cells, with a higher abundance than other amino acids. Glutamate occupies a central position in the amino acid metabolism network because it can affect the biosynthesis of proline, aspartate, alanine, and serine. Related genes such as GOT2, ALDH18A1, PYCR, and PSPH show an upregulated trend, but GPT, GLS, and GLUL do not show significant differences between regions. Aspartate is further used to generate asparagine, and ASNS enrichment can be observed in malignant regions, which is then used to generate arginine through the urea cycle. Serine provides methyl groups for single-carbon metabolism and generates glycine in the process. SHMT2 also shows an up-regulated trend. Serine can also be used in the trans-sulfurization pathway to generate cysteine (Figures 5A and 5B).

Polyamine metabolism is one of the important nitrogen-containing metabolic pathways, which plays a role in various malignant biological processes by regulating tumor cell metabolic flux and participating in epigenetic modification. Metabolites and enzymes related to polyamine metabolism show specific distribution in malignant regions, which may drive malignant progression. In the urea cycle (responsible for the upstream anabolism of polyamines), ARG1 and OTC are expressed at low levels, while ASS1 and ASL are the opposite. This shows the dependence of pancreatic cancer tissue on arginine. The increase in ornithine aminotransferase (OAT) suggests that the glutamate-proline metabolic pathway is a potential ornithine compensation pathway. Upregulated spermidine/spermine N1-acetyltransferase 1 (SAT1) and downregulated polyamine oxidase (PAOX) indicate that N^1^-acetylspermidine and N^1^-acetylspermine tend to be excreted from cells rather than replenishing spermidine and putrescine. At the same time, there are differences in spatial distribution and abundance between the two, suggesting that there are differences in demand (Figures 5C–5F).

### Integrated analysis of lipid metabolism and gene expression

**Figure 6:**
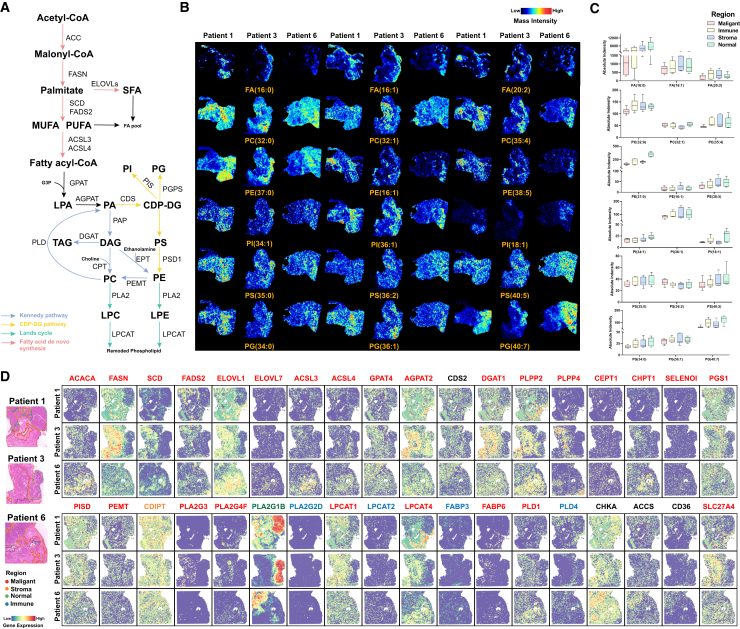
Visualization of metabolic reprogramming of lipid metabolism in pancreatic cancer (A) Schematic maps of lipid metabolism, including fatty acid *de novo* synthesis (red arrows), Kennedy pathway (blue arrows), CDP-DG pathway (yellow arrows), and Lands cycle (green arrows). (B) MS images of representative lipids in pancreatic cancer tissues (intensity in color scale is relative value). (C) Expression levels of representative lipids in different regions of pancreatic cancer tissue. (D) Spatial expression images of key genes in lipid metabolism (intensity in color scale is log2 transformed). Symbols: ns. denotes non-statistically significant, ∗ indicates a *p*-value <0.05, ∗∗ represents a *p*-value <0.01, and ∗∗∗ signifies a *p*-value <0.001. Data are represented as mean ± SEM.

The role of lipid metabolic reprogramming in tumors has been increasingly revealed (Figure 6A). ACC and FASN catalyze the *de novo* synthesis of fatty acids, which are then desaturated and elongated into different types of fatty acids under the action of SCD, FADS, and ELOVL. The above genes are highly expressed in malignant regions, but fatty acids are not significantly enriched in abundance. Phospholipids are basic components of cell membranes, and their metabolic reprogramming can participate in signal transduction. This study presents the spatial characteristics of different phospholipids, including phosphatidylethanolamine (PE), phosphatidylserine (PS) and phosphatidylinositol (PI), phosphatidic acid (PA), phosphatidylglycerol (PG), phosphatidylcholine (PC) and sphingomyelin (SM), etc. We found that although the content of these phospholipids is low, polyunsaturated long-chain phospholipids often have lower content in malignant regions (Figures 6B and 6C). Kennedy pathway and CDP-DG pathway are pathways for mutual conversion between phospholipids, and most of the genes in them are upregulated in malignant regions, suggesting active phospholipid remodeling. We then found that PLA2G3 and PLA2G4F were highly expressed in malignant regions, PLA2G1B in normal regions, and PLA2G2D in immune regions, suggesting the phospholipids heterogeneity between different regions (Figure 6D).

Cancer cells can absorb fatty acids through fatty acid transporters (FATPs), fatty acid translocases (CD36), and fatty acid binding proteins (FABPs). We found that CD36 was highly expressed in the immune regions, and FATP4 (also called SLC27A4) was highly expressed at the boundary between the malignant regions and the stromal regions, which may indicate their different metabolic requirements.

### Integrative analysis of metabolic differences between pathological regions

In addition to using scMetabolism to enrich pathways for different pathological partitions in the previous article, we also performed enrichment analysis based on DEGs and DEMs in ST and SM to find common differential pathways. Significant differences in pancreatic secretion and protein digestion and absorption pathways are evident between malignant tumor regions and normal tissues, indicating dysregulated pancreatic function in malignant areas. Enrichment analysis across multiple databases further confirmed notable variations in cell junction and cell adhesion pathways (Figure S25). Additionally, extracellular matrix (ECM)-related pathways differ between malignant tumor areas and stromal regions, potentially linked to the fibroproliferative TME characteristic of pancreatic cancer. Enrichment analyses also revealed dysregulated fatty acid production and transport, contributing to the lipid metabolism reprogramming observed, suggesting that stromal regions may supply lipid “fuel” to malignant areas (Figure S26). The immune region exhibited distinct differences in arachidonic acid metabolism and antigen processing and presentation pathways. Pathway enrichment analysis identified notable immune activity, including lysosome, ferroptosis, and phagosome-related pathways, indicating an immune response aimed at curbing the unchecked proliferation of cancer cells. Furthermore, cholesterol metabolism emerged from multi-omics enrichment analyses, warranting further investigation (Figure S27).

Using two specimens collected after neoadjuvant therapy, we explored the metabolic reprogramming of malignant and stromal regions post-treatment. Malignant areas displayed significant differences in glycolysis and pentose phosphate pathways, reflecting inhibited tumor proliferation. Interestingly, several metabolic pathways, such as central carbon metabolism in cancer, arginine and proline metabolism, protein digestion and absorption, and glycolysis/gluconeogenesis, showed consistent enrichment across the two specimens, highlighting shared gene/metabolite alterations. Enrichment analysis also suggested that angiogenesis and hypoxia responses might underlie the observed differences between the two groups (Figure S28). In stromal regions, differences in HIF-1 signaling were identified, consistent with hypoxia-driven fibrosis. Notably, treatment enhanced fatty acid metabolism, altered antigen processing and presentation pathways, and influenced immune responses (Figure S29) However, the underlying mechanisms require further detailed investigation.

## Discussion

Metabolic reprogramming is one of the important hallmarks of tumor cells, which can meet the synthesis of building blocks and energy components required for the malignant progression of cancer. However, PDAC often exhibits significant heterogeneity, and the complex communication between tumors and surrounding normal cells is also crucial for creating a metabolic microenvironment.^8^ Through ST sequencing based on 10× Genomics Visium and scRNA-seq annotation, we can accurately identify different tissue structures. MSI-based SM analysis can analyze metabolites in tissues *in situ* and perform spatially resolved mapping of PDAC-related metabolites. Many studies have revealed the metabolic changes that occur during the progression of pancreatic cancer from the perspective of transcriptomics or metabolomics. However, the spatial characteristics of metabolic reprogramming in the TME and the key molecular events in the malignant progression still need to be further explored. The integration of the ST and SM can provide a more comprehensive tumor metabolic landscape and help us visualize the complex process of tumor metabolic reprogramming.

In previous literature, SM based on DESI-MSI or MALDI-MSI has been carried out in various cancer studies,^9^ such as prostate cancer,^10^ breast cancer,^11^^,^^12^ lung cancer,^13^ colorectal cancer,^14^ and hepatocellular carcinoma.^15^ Although it is recognized that tumor cells have significant metabolic reprogramming, there is heterogeneity in the abundance of metabolites due to the functional characteristics of different tissues. Especially in pancreatic cancer, most metabolites may be present at lower levels in tumors than in normal tissues, which may be caused by the higher frequency of consumption than accumulation.^16^

Some previous studies have also explored the metabolomics characteristics of PDAC. For example, several studies have evaluated circulating metabolite biomarkers in serum,^17^^,^^18^^,^^19^ but they may be affected by metabolic-related confounding factors. Some studies explore the evaluation of surgical resection margins based on metabolites,^20^ or metabolomics analysis of patient-derived tumor xenografts (PDTX). However, there is still a lack of multi-omics research to explain the mechanism of metabolic reprogramming. Zhang et al. integrated metabolomics and transcriptomics to explain the inhibitory effect of fatty acids in PDAC^7^; Wang et al. conducted a very meaningful analysis from the perspective of lipidomics.^21^ With the development of spatial multi-omics technology and the improvement of sequencing accuracy, it has become possible to visualize metabolism in different tissue regions and even between different cells. At present, there is still a lack of in-depth research on the metabolic reprogramming process and prognostic characteristics of PDAC from the spatial multi-omics level.

Pancreatic acinar cells have the ability to dedifferentiate into ductal-like cells, ADM. Under abnormal conditions such as oncogene mutations, ductal-like cells continue to over-proliferate, forming pancreatic intraepithelial neoplasia (PanIN), which can further progress to PDAC.^22^^,^^23^^,^^24^ We used pseudotime analysis to explore metabolic changes in the evolution of pancreatic cancer. In the Pre-branch, acinar-based metabolic processes dominate, including fatty acid and amino acid degradation, while in branch 1, active extracellular matrix remodeling gradually emerges. In branch 2, tumor cells exhibit a completely different metabolic state, with enhanced metabolic activity that supports malignant proliferation, accompanied by enrichment of cell cycle and translation genes as well as endocytic activity. This suggests that during the progression from acinar to PDAC, significant metabolic remodeling occurs.^25^^,^^26^

To explore the metabolic heterogeneity of pancreatic cancer, scMetabolism, and SM enrichment analysis were performed to indicate metabolic activity in pancreatic tissue according to the defined pathological regions, and hypermetabolism and hypometabolism regions were distinguished. Because PDAC presents a complex TME, intercellular communication may help create this metabolic reprogramming. In the hypermetabolism region, although there was no significant increase in the overall number and intensity, a significant decrease in communication with astrocytes was found, accompanied by active communication with macrophages and fibroblasts.

To further explore the metabolic-immune microenvironment, we found that some ligand-receptor pairs were significantly enhanced in high-metabolism regions and correlated with prognosis. ADGRE5 (also called CD97) and its ligand CD55 are upregulated in PDAC,^27^ and function at cell-cell contacts, consistently being slightly upregulated in hypermetabolic regions. THBS1 and THBS2 are reported to be associated with ECM and angiogenesis inhibition processes in intrahepatic cholangiocarcinoma,^28^ and THBS1-related intercellular communication plays a role in liver fibrosis^29^ and contributes to immunosuppression and metastasis of colorectal cancer^30^; THBS2+ cancer-associated fibroblasts (CAFs) can promote the invasiveness of early lung adenocarcinoma.^31^ THY1 (also known as CD90) is a marker of fibroblasts,^32^ CAF-derived THY1 promotes metastasis through cross-talk within TME.^33^^,^^34^ COL1A1, COL1A2, and COL6A1 are related to type I collagen signaling and are significantly highly expressed in fibroblasts. Their interactions with SDC4, CD44, and various immune complexes are enhanced, shaping the immune barrier, preventing immune cell infiltration, and assisting immune evasion.^35^ AGRN and DAG1 have also been reported to be involved in ECM-receptor interactions in colorectal cancer.^36^ Secreted signaling is also an important cell-cell interaction. The AnxA1/FPR1 autocrine axis can promote invasiveness in breast cancer.^37^ Eph receptor-Ephrin signaling mechanism-mediated cell-cell communication and cell-autonomous signaling plays an important role in tumor cells and the TME.^38^ EFNA1-EPHA2 can promote breast cancer cell proliferation by increasing glutamine metabolism.^39^ Serum exosomal EphA2 protein is highly expressed in pancreatic cancer patients and is associated with poor prognosis.^40^ Hbegf-Egfr/Erbb2 is one of the main contributors to EGF signaling,^41^ and EGFR-activated myo-CAFs can promote pancreatic cancer metastasis.^42^ FN1-CD44 can play a role in bladder cancer drug resistance and glycolysis metabolic reprogramming,^43^ as well as cancer brain metastasis^44^ through the secretory protein pathway. Ductal cells in advanced PDAC are enriched in the mesenchymal marker FN1 and have high expression levels of the cancer stem cell (CSC)-associated gene CD44.^45^ The Gas6/AXL signaling pathway is closely related to the malignant behavior of tumor cells and can cause immunosuppression.^46^^,^^47^ Chemotherapy combined with Gas6/AXL targeted therapy may provide new hope for metastatic pancreatic cancer.^48^ GAS6-based CAR-T cells show effective anti-tumor activity.^49^ LGALS9-CD44 communication plays a role in the resilience of tumor-initiating cells (TICs) and the immunosuppressive microenvironment.^50^

In addition, we also performed spatial multi-omics visualization of different pathological regions to explore metabolic characteristics and diagnostic and therapeutic potential. Representative enriched metabolites can be identified in different pathological regions, such as glycocholic acid and PE (22:6) in normal regions, and ascladiol and 2-furoic acid in malignant regions, but further verification and mechanism exploration are still needed. Significant differences can also be observed in the metabolism of carbohydrates, amino acids, lipids, and nucleotides.

Cancer cells take up a lot of glucose, but also waste a lot of glucose and convert it into lactic acid for excretion,^51^ and a large amount of lactic acid will affect TME and promote tumorigenesis.^52^ In addition, cell-intrinsic programs drive immune cells and cancer cells to preferentially obtain glucose and glutamine, respectively,^53^ and cancer cells show stronger glutamine dependence.

PDAC can utilize glutamine to support its proliferation and redox balance, and therefore it is one of the most deficient amino acids in malignant regions,^54^ and therapies that inhibit glutamine metabolism have recently been explored.^55^ PDAC induces the expression of aspartate aminotransferase, which has also emerged as a promising therapeutic target.^56^ Glutamine is converted to glutamate by glutaminase (GLS), which in turn produces α-ketoglutarate that participates in the TCA cycle^57^ and, therefore, depletion in malignant regions can also be observed. Recently, it has been found that proline supports the production of extracellular matrix and contributes to tumor progression,^58^^,^^59^ and the proline metabolic limiting enzyme P5CS promotes the proliferation of PDAC.^60^ Although proline may be more dependent on pathways such as endocytosis for acquisition, active metabolic flux is also considered a promising target for intervention. Alanine was found to be lower in malignant regions,^61^ which is consistent with our results, and there is also lower GPT expression. It has also been shown that PSCs support tumor metabolism through autophagic alanine secretion.^62^ Tryptophan is an important step in one-carbon unit metabolism,^63^ and increased PSPH expression can be seen. PDAC cells show abnormal histidine uptake/accumulation, which leads to oxidative stress and amino acid pool depletion.^64^ Histamine is considered to be associated with inflammation, and significantly elevated metabolite levels can be observed in the stroma regions, especially at the junction of normal and malignant regions.

Polyamine metabolism is one of the important nitrogen-containing metabolic pathways in the human body, which widely affects various malignant biological processes such as cell survival and proliferation.^65^ By integrating ST and SM data, we found that the metabolites putrescine, ornithine and spermine, and related enzymes showed specific distribution in malignant regions, indicating abnormal polyamine metabolic reprogramming. We visualized the metabolic pathways of polyamine synthesis and degradation. In the upstream urea cycle, malignant regions showed arginine depletion,^66^ while the metabolic enzymes (ARG1/2, OTC) involved in the direct conversion of arginine and ornithine were expressed at a low level, while the metabolic enzymes involved in regulating aspartate (ASL, ASS1) were the opposite. This indicates that the overall metabolism of the urea cycle is relatively low, and PDAC may maintain ornithine content through other compensatory pathways. Based on previous studies, recent studies have found that this process is completed through the glutamate-proline metabolic pathway and may be assisted by OAT.^67^ Under physiological conditions, intracellular polyamines are mainly excreted in acetylated form, and a portion of acetylated polyamines can be oxidized to spermine or spermidine to maintain intracellular polyamine levels.^68^ The significant expression of SAT1 in malignant regions indicates enhanced acetylation of polyamines. At the same time, the expression level of PAOX is low, indicating that acetylspermine is mainly excreted from cells rather than replenishing spermidine levels through PAOX-dependent pathways. Although important metabolites in the salvage pathway were not identified, related enzymes (SRM, SMS) were still found to be upregulated in malignant regions, suggesting that there may be activation of the salvage pathway. In summary, our results indicate that PDAC has active polyamine metabolism and may have unique mechanisms.

The intricate roles of lipid metabolism reprogramming in cellular energy metabolism and cell signaling have been increasingly revealed.^69^ *De novo* synthesis of lipids is a metabolic source for tumor cell growth.^70^ Under the action of ACC and FASN, acetyl-CoA synthesizes palmitic acid (FA-16:0), and under the action of SCD, FADS, and ELOVL it can continue to desaturate and extend into different types of fatty acids. We show here that although the above-mentioned key genes are significantly enriched in malignant regions, there is no enrichment of free fatty acids. Zhang et al. also found this phenomenon, that is, there is a serious lipolysis network disorder in PDAC, and palmitic acid and stearic acid are significant, which plays a tumor suppressor role by inducing cell apoptosis.^7^ Some unsaturated FFAs are also upregulated in immune regions, accompanied by a relative increase in FADS and ELOVL in immune regions, indicating that it may be related to immune response. Phospholipids are basic components of cell membranes. Phospholipid metabolic reprogramming may play a role in biological membrane synthesis and signal transduction,^71^ and has diagnostic and therapeutic potential in pancreatic cancer.^21^ Kennedy pathway and CDP-DG pathway constitute a metabolic network for synthesizing phospholipids using PA and triglycerides (TG).^72^ Although CDS2 did not show significant regional characteristics, most of the subsequent genes were upregulated in the malignant region, suggesting significant phospholipid metabolic reprogramming. Interestingly, although the abundance of phospholipids in the malignant region was not high, polyunsaturated phospholipids seemed to be distributed less in the malignant region. This suggests that it may be to avoid ferroptosis caused by phospholipid peroxidation composed of polyunsaturated fatty acids.^73^^,^^74^ We then further focused on the Lands cycle that affects lipid membrane remodeling. PLA2 and LPCAT family genes showed significant regional specificity. PLA2G1B and pancreatic acinar cell digestion of dietary phospholipids,^75^ PLA2G2D and anti-inflammation and immunosuppression,^76^ PLA2G4F and membrane lipid remodeling^77^ are consistent with their spatial localization. LPCATs also showed different enrichment characteristics, among which LPCAT1 and LPCAT4 controlled membrane phospholipid saturation and sustained proliferation signals, and are expected to become new targets.^78^ In addition, FATPs, CD36, and FABPs are responsible for absorbing exogenous fatty acids and giving cancer cells metabolic flexibility.^79^ At the same time, we also noticed that a variety of lipid metabolites were significantly enriched in the stroma region. Auciello et al. found that PSCs were activated into a fibroblast phenotype and underwent lipid metabolism reprogramming, including downregulation of lipid storage-related genes and massive lipid secretion, as a potential fuel source for PDAC cells, and identified the autotaxin-LPA axis to promote PDAC cell progression.^80^ Combined with the extensive cell communication between PDAC cells and fibroblasts, as well as the spatial localization of related genes, and the possible loss of lipids during PSC transformation, we hypothesize that CAFs may be an important provider of lipids for cancer cells, but further experimental confirmation is still needed. At the same time, our research group also conducted plasma lipidomics sequencing of pancreatic cancer and control groups (unpublished), and similar lipid metabolism reprogramming phenomena were observed.

In addition, we also imaged some other metabolites. For example, malignant regions have higher dopamine and traumatic acid, and less ascorbic acid, which may be related to dopamine receptors,^81^^,^^82^ sarcopenia,^83^ and resistance to oxidative stress.^84^^,^^85^ Similar to this article, elevated taurine content was found in early pancreatic cancer metabolomics.^86^ Recent studies have found that tumor cells can overexpress SLC6A6 to compete for taurine, thereby inducing T cell death and dysfunction and promoting tumor progression^87^; in addition, taurine secreted by TAM can inhibit ferroptosis in prostate cancer by activating the liver X receptor α/stearoyl-CoA desaturase 1 pathway.^88^ Penet et al. found that the content of choline compounds in PDAC cell lines and tumors was increased,^89^ which may be related to the synthesis of phosphatidylcholine. Inhibition of choline transporter can play an anti-cancer role.^90^ In addition, 11C-choline PET/CT was used to evaluate recurrent prostate cancer.^91^ However, the role and mechanism of the above metabolites need to be further explored.

We also demonstrated differences in pathway enrichment between different regions based on pathological regions, and confirmed the above-mentioned metabolic reprogramming process, which is expected to help with early diagnosis and mechanism exploration. At the same time, understanding the metabolic reprogramming induced by neoadjuvant chemotherapy for pancreatic cancer is also expected to promote anti-tumor immunity and chemotherapy response.^92^ Similarly, there are significant differences in glycolysis in malignant regions, significant differences in complement pathways and antigen presentation in stroma regions, and common metabolic changes in lipid metabolism. This suggests that intervention measures targeting the above-mentioned pathways are expected to provide new ideas for sensitizing anti-tumor therapy.

This spatial multi-omics study on the metabolic reprogramming of PDAC provides a comprehensive visualization of metabolic heterogeneity within tumor tissues at the cellular level. It further elucidates distinct ligand–receptor communication patterns and identifies key regulatory factors across different metabolic regions, offering insights into their potential impact on disease prognosis. Multi-omics visualization was performed at the level of amino acid metabolism and lipid metabolism, revealing the heterogeneity of metabolomics and transcriptomics. At the same time, multi-omics differences and enrichment analysis of different pathological subregions, as well as insights into cancer-related metabolic dependencies and immune metabolic changes, are expected to better reveal the molecular mechanisms of tumors and provide new ideas for targeted clinical diagnosis and treatment.

### Limitations of the study

Unfortunately, the accuracy of the study is hampered by the limited spatial omics resolution and the lack of fine annotation of single cells. In addition, *in vivo* and *in vitro* experiments are still needed to verify the intrinsic mechanism. At the same time, with the progress of spatial proteomics and epigenetics, a more complete multi-omics analysis may better explain biological hypotheses. Finally, the inclusion of healthy control samples, along with the application of machine learning and other advanced technologies, may offer new insights into the differences between various pathological regions. Additionally, future research should focus on large-scale studies that minimize the influence of factors such as diet and treatment on metabolic pathways, while striving to integrate and clarify the changes and regulatory mechanisms within the gene-protein-metabolite axis.

### Conclusions

In summary, by integrating ST and SM, we can accurately image the metabolic differences between different pathological divisions of PDAC, visualize the abnormal metabolic processes, and further associate them with different metabolic pathways. Further transcriptome analysis reveals the metabolic changes and interactions during the progression of PDAC and explores its role in malignant progression, providing new clues for targeted clinical treatment.

## Resource availability

### Lead contact

Further information and requests for resources and reagents should be directed to and will be fulfilled by the lead contact, Ziwen Liu (liuziwen@pumch.cn).

### Materials availability

This study did not generate new unique reagents.

### Data and code availability

• The raw sequence data of spatial transcriptome reported in this paper have been deposited in the Genome Sequence Archive (Genomics, Proteomics & Bioinformatics 2021) in National Genomics Data Center (Nucleic Acids Res 2021), China National Center for Bioinformation/Beijing Institute of Genomics, Chinese Academy of Sciences (Genome Sequence Archive for Human: HRA009976; Project: PRJCA034193) that are publicly accessible at https://ngdc.cncb.ac.cn/gsa-human.
  The raw sequence data of spatial metabolome reported in this paper have been deposited in the METASPACE, the platform for metabolite annotation of imaging mass spectrometry data, which are publicly accessible at https://metaspace2020.eu/project/liu-2025. At the same time, we also uploaded the relevant information to the MetaboLights database (Study:MTBLS12204, https://www.ebi.ac.uk/metabolights/MTBLS12204) according to the Cell Press approved standardized repository.
• This paper does not report original code.
• Any additional information required to reanalyze the data reported in this paper is available from the lead contact upon request.

## Acknowledgments

The authors thank Shanghai OE Biotech CO., Ltd. and Shanghai Luming Biological Technology Co., Ltd. for the access to the 10× Genomics Visium and AFADESI-MSI platforms.

Supported by the 10.13039/501100001809National Nature Science Foundation of China (nos. 82172727 and 81572459), Nature Science Foundation of Beijing (nos.7202164), CAMS Innovation Fund for Medical Sciences (CIFMS) (no.2024-I2M-ZD-001), and National High Level Hospital Clinical Research Funding (no. 2022-PUMCH-D-001).

## Author contributions

All authors assisted in conducting the study. H.W. and Z.L. participated in the conception and design; Z.C. and X.C. participated in the pathological analysis; Q.Z. and X.H. participated in the acquisition and preparation of specimens; H.W. and Q.Z. participated in drafting the paper or revised the content of the paper critically; H.C. and M.W. assisted in logical consistency and language polishing; M.F. and T.H. assisted in creating and revising figures; X.H., X.C., and Z.L. participated in reviewing the final version for publication. All authors agree to be responsible for all aspects of the work.

## Declaration of interests

The authors declare that they have no competing interests.

## STAR★Methods

### Key resources table

**Table:** 

REAGENT or RESOURCE	SOURCE	IDENTIFIER
**Biological samples**		

Pancreatic cancer tissues	Peking Union Medical College Hospital	N/A

**Critical commercial assays**		

10x Genomics Visium CytAssist based spatial transcriptomic detection	Shanghai OE Biotech CO., Ltd.	N/A
AFADESI-MSI based spatial metabolomics detection	Shanghai Luming Biological Technology Co., Ltd.	N/A

**Software and algorithms**		

cellSens Dimension Software	Olympus	N/A
10x Genomics Visium library preparation protocol	10x Genomics	200100
Space Ranger	10x Genomics	N/A
SVD	OmniAnalyzer Pro	N/A
Uniform manifold approximation and projection (UMAP)	OmniAnalyzer Pro	N/A
Seurat	OmniAnalyzer Pro	N/A
Inferred copy number variation (inferCNV)	OmniAnalyzer Pro	N/A
Diffusion pseudotime (DPT)	OmniAnalyzer Pro	N/A
Partition-based graph abstraction (PAGA)	OmniAnalyzer Pro	N/A
Differentially expressed gene (DEG)	OmniAnalyzer Pro	N/A
R software (version 4.3.1)	the R Core Team and the R Foundation for Statistical Computing	https://www.r-project.org/
clusterProfiler	R package	https://bioconductor.org/packages/release/bioc/html/clusterProfiler.html
enrichplot	R package	https://bioconductor.org/packages/release/bioc/html/enrichplot.html
Limma	R package	https://bioconductor.org/packages/release/bioc/html/limma.html
reshape2	R package	https://cran.r-project.org/web/packages/reshape2/index.html
Ggpurb	R package	https://cran.r-project.org/web/packages/ggpubr/index.html
Survival	R package	https://cran.r-project.org/web/packages/survival/index.html
survminer	R package	https://cran.r-project.org/web/packages/survminer/index.html
Cardinal	R package	https://bioconductor.org/packages/release/bioc/html/Cardinal.html
Ropls	R package	https://bioconductor.org/packages/release/bioc/html/ropls.html
Pheatmap	R package	https://www.rdocumentation.org/packages/pheatmap/versions/1.0.12/topics/pheatmap
RCTD	R package	https://github.com/dmcable/spacexr
addmodulescore	R package	https://www.rdocumentation.org/packages/Seurat/versions/2.3.4/topics/AddModuleScore
scMetabolism	R package	https://github.com/wu-yc/scMetabolism
ggplot2	R package	https://ggplot2.tidyverse.org/reference/

**Deposited data**		

The Cancer Genome Atlas (TCGA)	the TCGA data portal	https://portal.gdc.cancer.gov/
Spatial transcriptome data	Genome Sequence Archive for Human	HRA009976 (Project: PRJCA034193)
Spatial metabolome data	Metabolights	https://www.ebi.ac.uk/metabolights/MTBLS12204
Spatial metabolome data	METASPACE	https://metaspace2020.eu/project/liu-2025

### Experimental model and study participant details

#### Ethics approval and consent to participate

All relevant procedures were approved by the Institutional Review Board (IRB). We designed this study in compliance with the Helsinki Declaration and Good Clinical Practice and approved by the Ethnical Committee of Peking Union Medical College Hospital (No. I-23PJ941). Informed written consent was obtained from all participants.

#### Human pancreatic cancer tissue specimen

A total of six postoperative cancer tissue from patients diagnosed with pancreatic cancer and underwent surgery at Peking Union Medical College Hospital were included in this study. All the tumor classification of these patients is pancreatic ductal adenocarcinoma (PDAC). Diagnoses of all specimens were confirmed by two senior pathologists according to the histopathological examination. TNM and clinical stages were defined in accordance with the eighth edition of the American Joint Committee on Cancer (AJCC) staging system. Fresh samples were embedded in pre-chilled optimal cutting temperature (OCT) compound after drying and snap frozen in dry ice and then stored at −80°C refrigerator. the study does not involve animal models, cell lines, primary cell cultures, or clinical trials. Detailed information about the samples of included patients can be found in Table S1.

### Method details

#### Preparation and processing of cancer tissue section

The postoperative PDAC tissues were embedded in OTC and cut into 10 μm serial frozen sections at −20°C on a cryostatmicrotome (Leica CM 1860 UV). One set of tissue sections were mounted onto Superfrost Plus slides (Thermo Fisher) for AFADESI-MSI to enable spatial metabolomics analysis. Before AFADESI-MSI analysis, the tissue sections were dried in vacuum for about 15min. Another set of tissue sections were stained with hematoxylin and eosin and evaluated by pathologists to selected a 6.5 × 6.5mm area with the most significant tumor heterogeneity from the entire tissue section. Then, the original tissue samples were cut according to the rectangular area delineated by the pathologist mounted onto 10 × Genomics Visium array slides for spatially resolved transcriptomics analysis.

#### Spatially resolved transcriptomics analysis

Slide Preparation, Fixation and Staining: Tissue samples were prepared and processed based on the User Guide of Visium CytAssist Spatial Gene Expression Reagent Kits (CG000495). Frozen tissue sections (10 μm, adjacent to the ones being analyzed by AFADESI-MSI) were mounted onto 10×Visium spatial slides printed with four identical capture areas, each with about 5000 unique gene expression spots. Each spot on the slide was approximately 55 μm in diameter, with six spots nearby, and the distance between adjacent array spots is 100 μm. Fixation, H&E staining, and imaging were performed based on the Methanol Fixation, H&E Staining & Imaging for Visium Spatial Protocols (CG000160). Sectioned slides were incubated in precooled (at −20°C) methanol (Millipore Sigma, Darmstadt, Germany) and isopropanol (Millipore Sigma). Then at room temperature, these slides were incubated in Mayer’s hematoxylin (Dako, Agilent, Santa Clara, CA) for 7 min, stained in Bluing loading buffer (Dako) for 2 min, and Eosin (Millipore Sigma) diluted 1:10 in Trisbase (ThermoFisher Scientific, 0.45 m, pH 6.0) for 1 min. After each of the staining steps, slides were washed in Milli-Q water (Corning, Corning, NY). Next, the slides were mounted in 80% glycerol and brightfield images were taken via 3D HISTECH (3DHISTECH Ltd., Budapest, Hungary).

Permeabilization, Reverse Transcription, Spatial Library Construction, and Sequencing: After fixation and staining, the issue sections were permeabilized using permeabilization enzyme (10× Genomics, 70 μL per well) .The slides were incubated at 37°C for different times and then washed in 100 μL 0.1× SSC (Sigma–Aldrich, St. Louis, MO) after removing permeabilizing enzymes. Poly adenylated mRNA released from the overlying cells were captured by specific probes on the slides. Master Mix containing reverse transcription (RT) reagents and fluorescently labeled nucleotides were added to obtain fluorescently labeled cDNA. After removing excess tissue, fluorescently labeled cDNA covalently linked to oligonucleotides were left and visualized. The optimal permeabilization time for this procedure was determined by the intensity of the fluorescent signal. Tissue sections were permeabilized using permeabilizing enzymes at optimal permeabilization times to release mRNA from cells in the tissue sections. Reverse transcription was performed on PCR instrument (MyCycler, Bio-Rad, Hercules, CA) following Visium Spatial Gene Expression Reagent Kits – Tissue Optimization User Guide (CG000238). After reverse transcription, cDNAs in the supernatant layer were collected for synthesis, inactivation, *in vitro* transcription, and adaptor ligation, the products from which were used in a second reverse transcription for the construction of the spatial library. After transfer of cDNA fromthe slide, spatially barcoded, full-length cDNA is amplified by PCR to generate sufficient mass for library construction. Then, P5, P7, i7, and i5 sample indexes, and Tru- Seq Read 2 are added via End Repair, A-tailing, Adaptor Ligation, and PCR. The final libraries contain the P5 and P7 primers used in Illumina amplification. TruSeq Read 1 is used for priming and sequencing the 16 bp Spatial Barcode and 12 bp UMI, and TruSeq Read 2 is used for priming and sequencing the cDNA insert. The two 10 bp sample indexes are sequenced in the i5 and i7 read respectively. Sequencing of the spatial library was performed on the Illumina NovaSeq 6000 platform.

Data Processing: First, the quality control of FASTQ files was evaluated using FastQC software. Space Ranger Soteware (Version 2.0.1) was used to process Visium ST sequencing data and brightfield microscope images. Based on the spatial barcode information, total spots, total reads per spot, counts, and unique molecular identifiers (UMIs) were evaluated for quality; The STAR software integrates the reference genome (GRCh38 human) for comparison analysis and generates a gene-spot matrix for gene expression analysis. After preliminary quality control, the Seurat software package (Version 4.3.0) was used for processing, scTransform was used for normalization and detection of high variance features (HVGs). R package Harmony was used to correct the batch effect of expression profiles. Principal component analysis (PCA) was used for linear dimensionality reduction, and unsupervised clustering and two-dimensional spatial visualization were performed using FindNeighbors, FindClusters, and RunUMAP Seurat functions. Based on the 16 clusters obtained by unsupervised cluster analysis, FindAllMarkers in the Seurat package performed differential expression analysis to find potential marker genes for each cluster. The identified marker genes were visualized with the help of VlnPlot and FeaturePlot functions. The significantly different genes with a p value less than 0.05 and a difference multiple greater than 1.5 were screened out, and GO and KEGG enrichment analysis was performed based on the hypergeometric distribution.

Cell Type Identification: RCTD (version 1.1.0) was used to infer the composition of cell types in each spot site. Using SPOTlight, our center's previous single-cell RNA-seq (PRJCA001063) was used as a reference dataset and mapped to this file. NormalizeData, ScaleData Seurat, RunPCA, FindNeighbors, FindClusters, RunUMAP, singleR, and FindAllMarkers Seurat functions were used to preprocess, reduce dimensions, cluster, visualize, and annotate cell types. The spotlight_deconvolution SPOTlight function based on non-negative matrix factorization (NMF) was used to deconvolute the ST data, and the spatial_scatterpie function was used to visualize the proportion of cell types in each spot.

Pseudotime Analysis: Through the Monocle package, machine learning was performed based on the expression patterns of key genes in the malignant progression of acinar cells and ductal cells. First, genes with large gene expression variation between cells were selected, and spatial dimensionality reduction was performed based on their expression profiles. Then, a minimum spanning tree (MST) was constructed. Based on the characteristics of the malignant trajectory, a branch expression analysis model (BEAM) was established to simulate the differentiation trajectory and dynamic changes of cells with similar transcriptional characteristics during the temporal development process.

Metabolism signature enrichment analysis: Metabolism signature enrichment analysis for spots of ST data was performed using the scMetabolism algorithm. scMetabolism is designed to easily quantify single cell metabolic activities by using a single line of commands. The core functionality of scMetabolism is to quantify metabolic pathway gene sets. Published gene sets and manually curated gene sets from the KEGG database and REACTOME database are combined to generate metabolic gene set lists. Metabolic activities can be quantified and correlation analysis performed with the raw data matrix using VISION, AUCell, and ssGSEA.

Transcription Factor Analysis: SCENIC was used to analyze the transcription factors (TFs) and their regulation between high-metabolism and low-metabolism regions. GRNBoost2 was used to construct the co-expression module (regulon) of TF and potential target genes. The regulon activity score (RAS), regulon specificity score (RSS), and connection specificity index (CSI) were calculated to represent the regulon activity score of each cell, the specific correspondence between the regulon and each cell type, and the correlation between different regulons.

Ligand–Receptor Interaction Analysis: The ligand-receptor interaction network between macrophages, fibroblasts, ductal cell type 2, and T cells was inferred by Cellchat. First, the expression and interaction of ligand-receptor pairs were obtained. The cell interaction network was imaged using the R packages igraph and qgraph, and the number of ligand-receptor pairs was recorded. The dot plots of differentially expressed ligand-receptor pairs were visualized using the R package ggplot2. Finally, the spatial expression of ligand-receptor pairs was presented using the SpatialDimPlot Seurat function.

#### Spatially resolved metabolomics analysis

Slide Preparation and Detection: Fixed tissue samples were placed in a Cryostat microtome (Leica CM 1950, Leica Microsystems, Germany) and sliced at a thickness of 10 μm and mounted on Superfrost Plus positive charge ionizing microscope slide (Thermo Fisher). H&E staining was performed on adjacent serial sections to annotate the histological regions. After being taken out of the −80°C ultra-low temperature freezer, the frozen tissue sections were quickly dried in a vacuum desiccator at room temperature for about 30 min. AFADESI-MSI analysis was conducted as described in previously published research. The AFADESI-MSI platform (Beijing Victor Technology Co., Ltd., Beijing, China) was equipped with a Q-Exactive Orbitrap mass spectrometer (Thermo Scientific, Bremen, Germany) and an AFADESI ion source. The spray solvent used in this study was acetonitrile and water (80:20, v/v), and the flow rate of the spray solvent was set at 2.0 μL/min. The experiments were performed in positive and negative ion modes at m/z 70–1200 with a resolution of 20 000 Daltons. The flow rate of the nebulizing gas was set at 0.6 MPa. The distance from sprayer to surface was 3 mm and the spray angle was 60°. The imaging analysis was performed by continuously scanning the tissue slices at a speed of 100 μm/sec in the x direction and at intervals of 100 μm in the y direction. Data processing was performed using MassLynx data acquisition and HDI processing systems.

Data Processing and Metabolites Annotation: The raw data (.raw files) was converted to. imzML files by imzMLConverter processing software and imported into Cardinal software for background subtraction, peak alignment, and peak screening. Subsequently, the corresponding overlap between MSI and H&E histological images can be constructed by the open software MSiReader (MSI Software Solutions, LLC, North Carolina, USA) based on the Matlab platform, and different tissue regions can be annotated. The correlation model of metabolites in different tissue regions was constructed via OPLS-DA and the contribution of metabolites to the differences between tissue regions was evaluated using the variable importance of projection (VIP) value. After averaging the m/z intensity of each pixel, the expression intensity of the corresponding mass-charge was displayed using the mass spectrum. The adduct ions extracted by AFADESI-MSI detection were annotated using the dedicated spatial metabolome smetDB database (Shanghai Luming Biotechnology Co., Ltd.) and the pySM annotation framework. Metabolite information was compared with the public databases Human Metabolome Database (HMDB) (https://hmdb.ca/), Metlin (https://metlin.scripps.edu/), LIPID MAPS (https://www.lipidmaps.org/), KEGG (https://www.kegg.jp/kegg), ChEBI (https://www.ebi.ac.uk/chebi/), and PubChem (https://pubchem.ncbi.nlm.nih.gov/).All annotated metabolites had m/z ratios less than 10 ppm (parts per million) compared to their monoisotopic molecular weights (MMW) and detailed information concerning the scanned m/z is shown in Table S1 (supplemental information). All annotated metabolites had m/z ratios less than 10 ppm (parts per million) compared to the monoisotopic molecular weight (MMW). Two-tailed Student's t-tests were performed to confirm the statistical significance of DEMs between tissue regions. Among them, VIP > 1.0, |log2(FC) > 1| and p value < 0.05 were defined as the criteria of DEMs. Metabolic set variation analysis (MSVA) is a non-parametric, unsupervised analysis method that is mainly used to evaluate the enrichment results of chips and metabolomes, using the KEGG database and Reactome database.

### Quantification and statistical analysis

We used SPSS 26.0 (IBM SPSS Statistics, Armonk, NY: IBM Corp), R software version 4.3.1 (The R Foundation for Statistical Computing, Vienna, Austria), GraphPad Prism 8.0 (GraphPad Software, Boston). Raw data from ST were log2 transformed and normalized for further analysis. Data in this study are presented as mean ± SD, and the sample size for each statistical analysis was at least 3. Mann–Whitney U-test was utilized to analyze continuous variables, which are presented as mean ± standard deviation (SD) in some conditions. Statistical significance would be considered when P < 0.05.

## Supplemental information

Document S1. Figures S1–S29 and Tables S1 and S4

Table S2. Annotated metabolites among the metabolic pathways analyzed in this study

Table S3. Representative differentially enriched metabolites in pathological subregions of spatial metabolomics
