# Supplementary material for: Integrated spatial omics of metabolic reprogramming and the tumor microenvironment in pancreatic cancer
Source: iScience. 2025 May 15;28(6):112681. doi: 10.1016/j.isci.2025.112681 (PMC12177182; doi:10.1016/j.isci.2025.112681)
Supplement: Document S1. Figures S1–S29 and Tables S1 and S4 [file mmc1.pdf]

iScience, Volume *28*

Supplemental information

Integrated spatial omics of metabolic reprogramming and the tumor microenvironment in pancreatic cancer

Hao Wu (吴昊), Qiyao Zhang (张起尧), Zhen Cao (曹桢), Hongtao Cao (曹洪滔), Mengwei Wu (吴孟闱), Mengdi Fu (付梦迪), Tingping Huang (黄婷萍), Xianlin Han (韩显林), Xiaoyan Chang (常晓燕), and Ziwen Liu (刘子文)

Figure S1 Spatial transcriptomic characteristics of six PDAC samples.

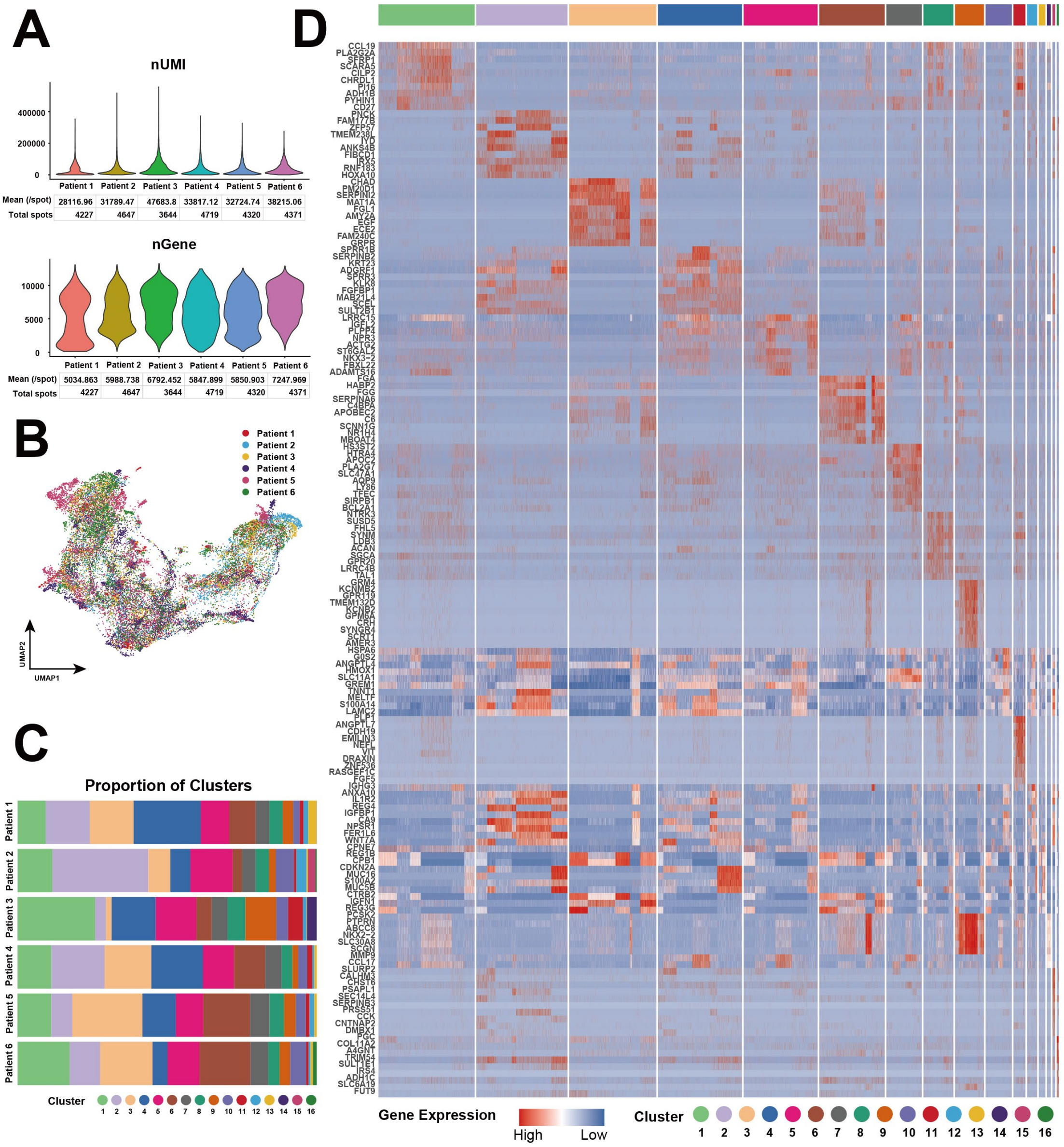

A: Violin plots displaying the average numbers of unique molecular identifiers (nUMI, top) and gene expression (nGene, bottom) in each spot of 6 samples. B: 25,928 spots obtained from ST were performed unsupervised clustering analysis UMAP and the distribution of each spot in the UMAP plot was colored by patients. There was a balanced distribution among spots from 6 samples, indicating the absence of batch effect. C: Bar plots showing the proportion of 16 clusters (obtained from the UMAP analysis) in each patient. D: Top 10 representative marker genes of each cluster were obtained through differential expressed gene (DEG) analysis. Heatmap showed the expression patterns of the top 10 marker genes in each cluster

Figure S2 Histological examination of six PDAC samples.

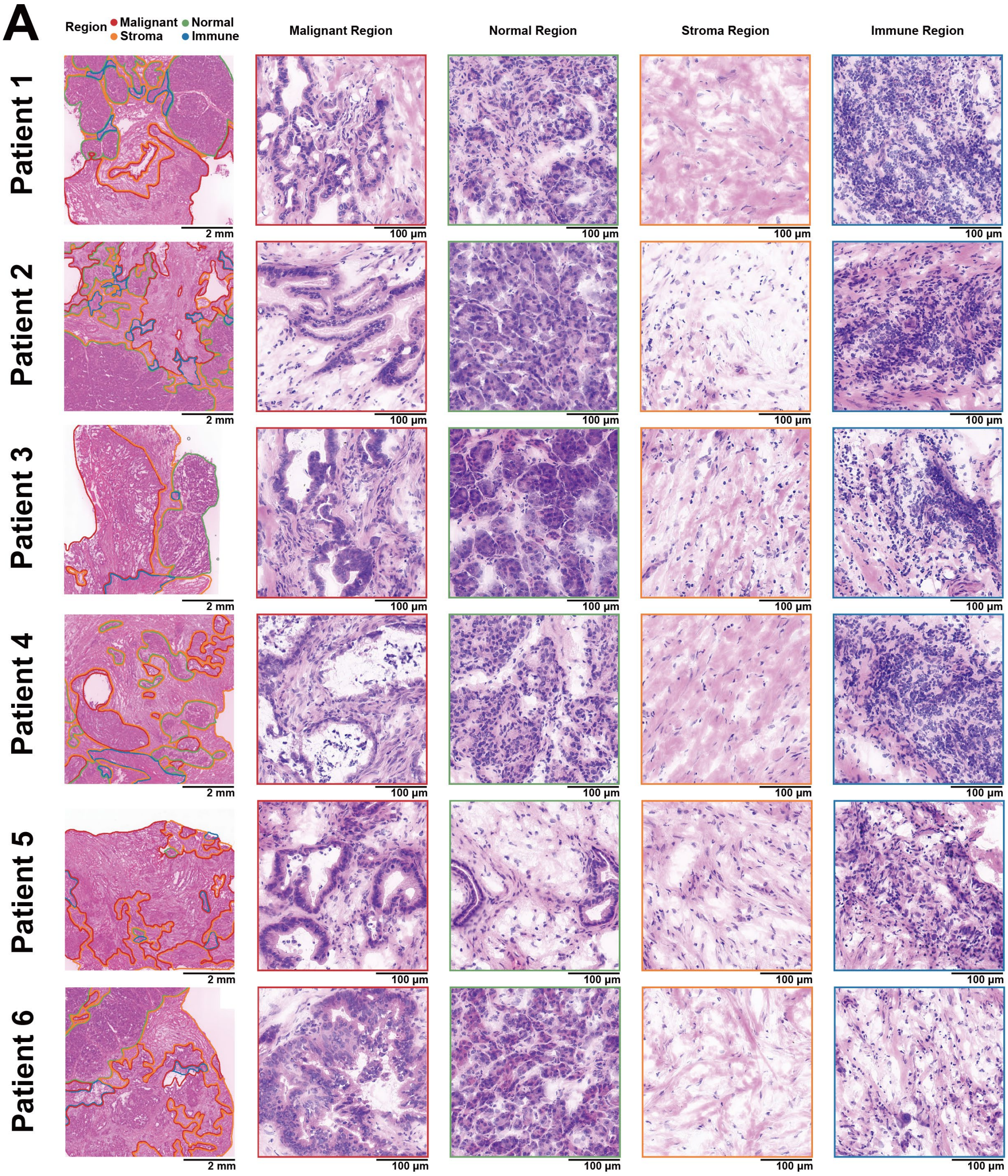

A: After H&E staining, representative fields (6.5 mm × 6.5 mm) of six PDAC samples were selected for spatial transcriptomic and metabolomic analysis in accordance with the guidance of senior pathologists. There are Malignat (red line surrounded), Normal (green line), Stroma (orange line), Immune (brown line) regions in the 6 samples. Representative enlarged views of certain histopathological regions were presented with scale bars of 100 μm.

Figure S3 Spatial distribution of representative marker genes.

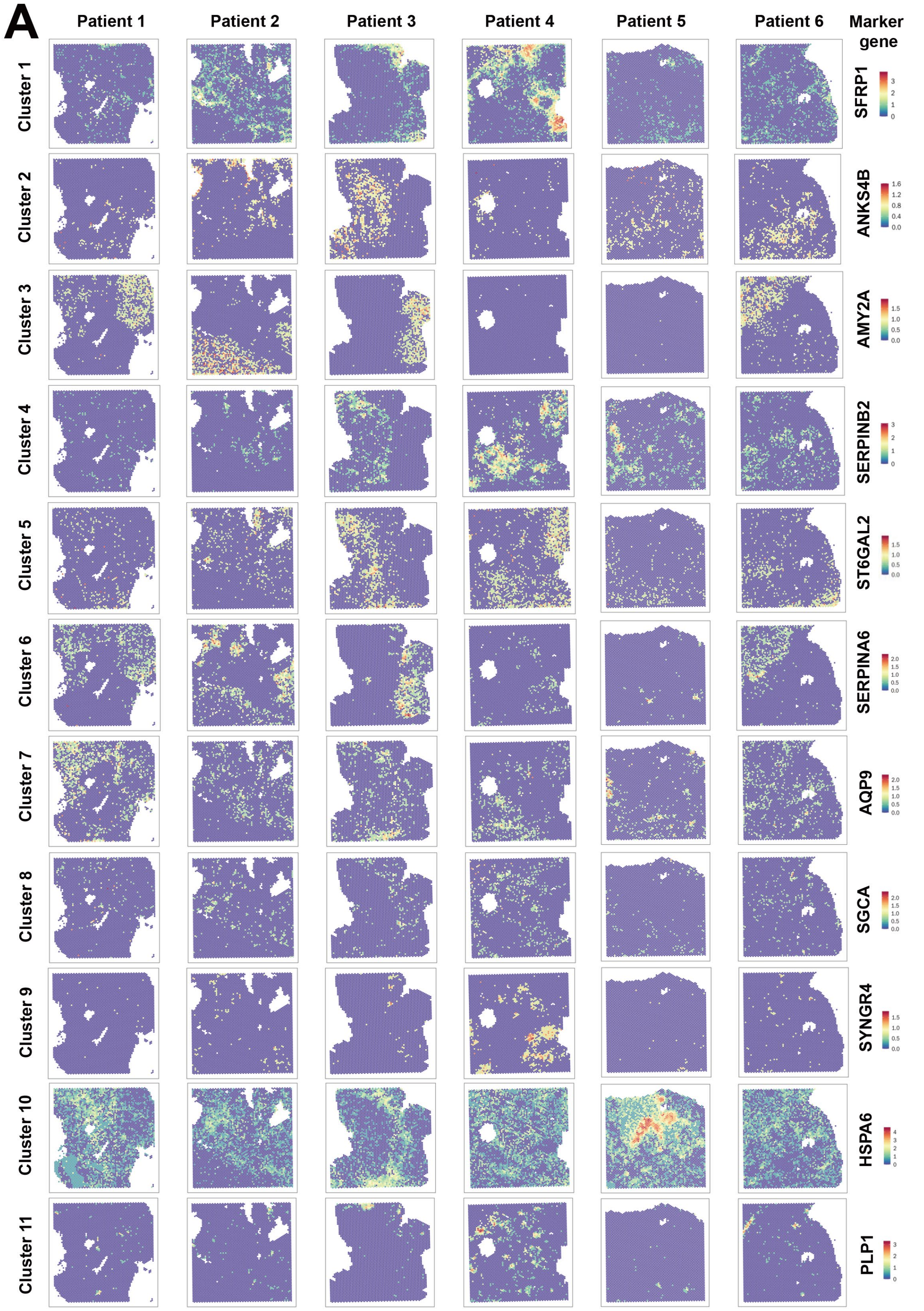

A: Spatial transcriptomic feature plots showing the spatial distribution of representative marker genes of each cluster in PDAC samples.

Figure S4 Cell constitution analysis of each spot in six samples.

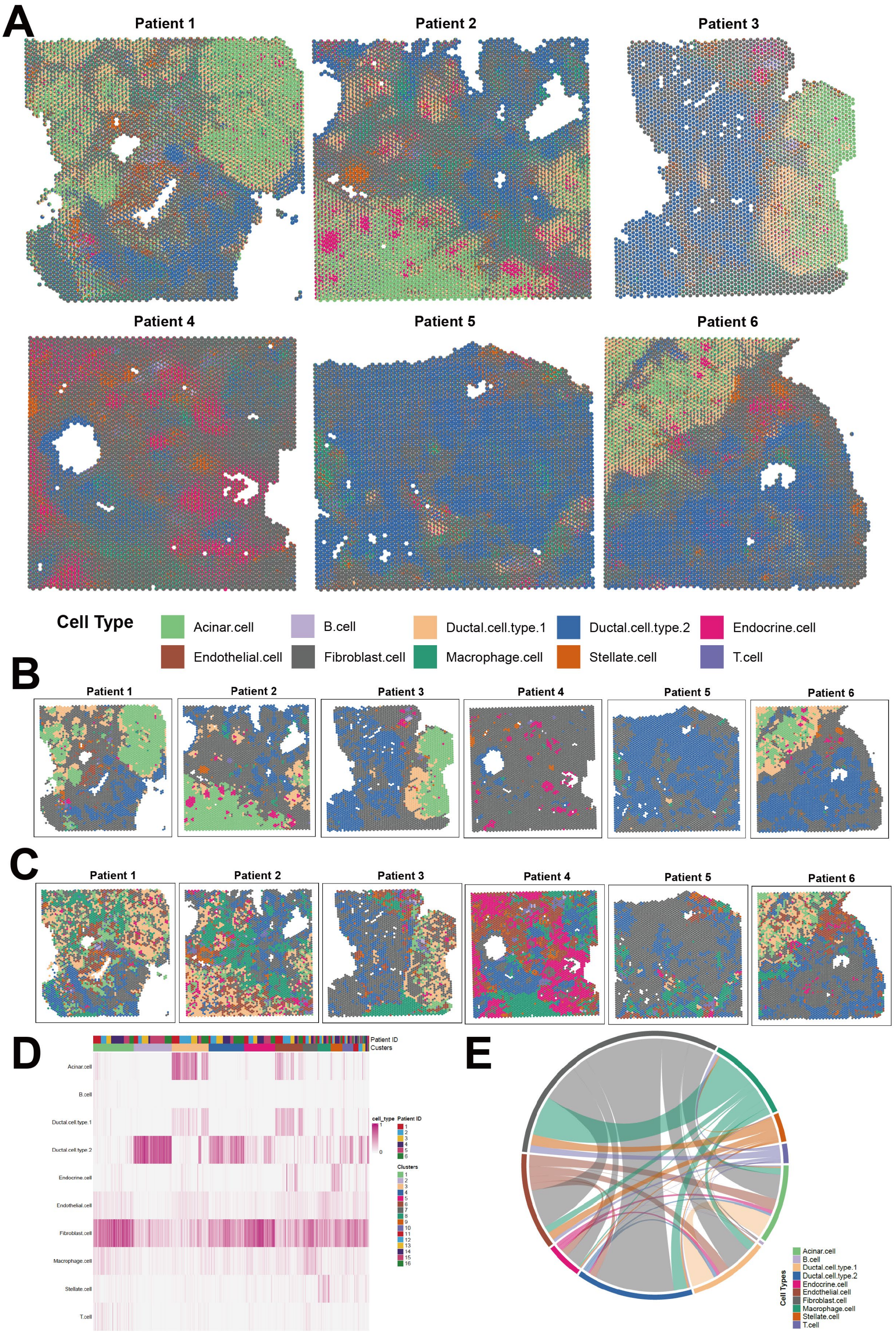

A: Given the limited resolution and complex cell constitution in one spot, we integrated a public

scRNA-seq data (PRJCA001063). SPOTlight was performed to analyze the constitution and proportion of annotated cell types in each spot of 6 PDAC samples. The constitution of cells included Aciar cell, B cell, Ductal cell type 1, Ductal cell type 2, Endothelial cell, Fibroblast cell, Macrophage cell, Stellate cell, and T cell. B-C: According to the proportion of different cell types in each spot, the cell type with the highest proportion (B) and the second highest proportion (C) are shown. D: Heatmap of different cell types in each cluster and sample. E: Heatmap of different cell types in each cluster and sample.

Figure S5 Mapping distribution of different cell types on tissue sections

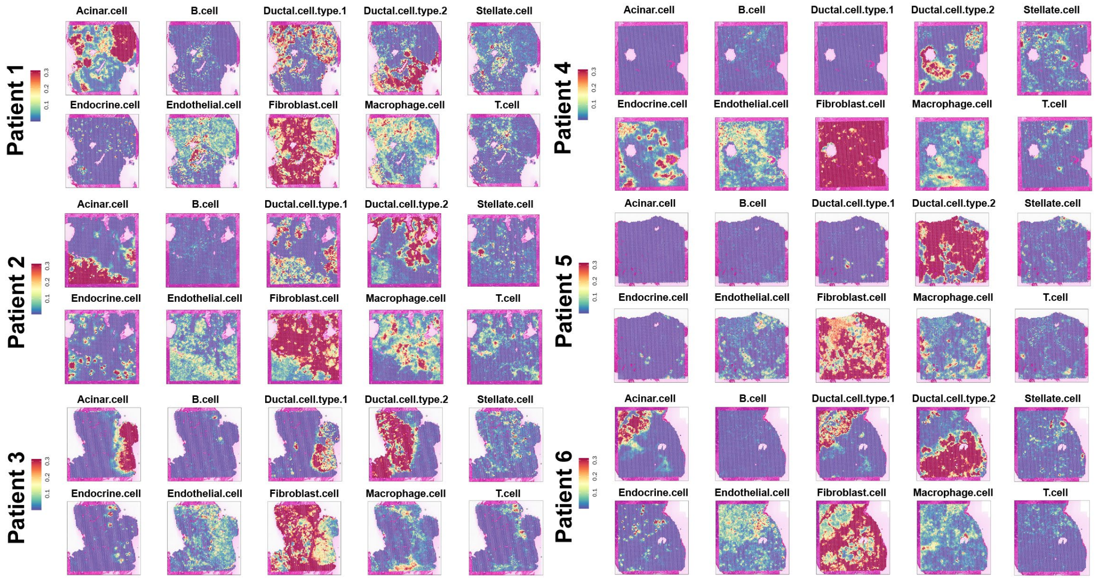

Figure S6 Spatio-temporal evolution and cell communication in PDAC.

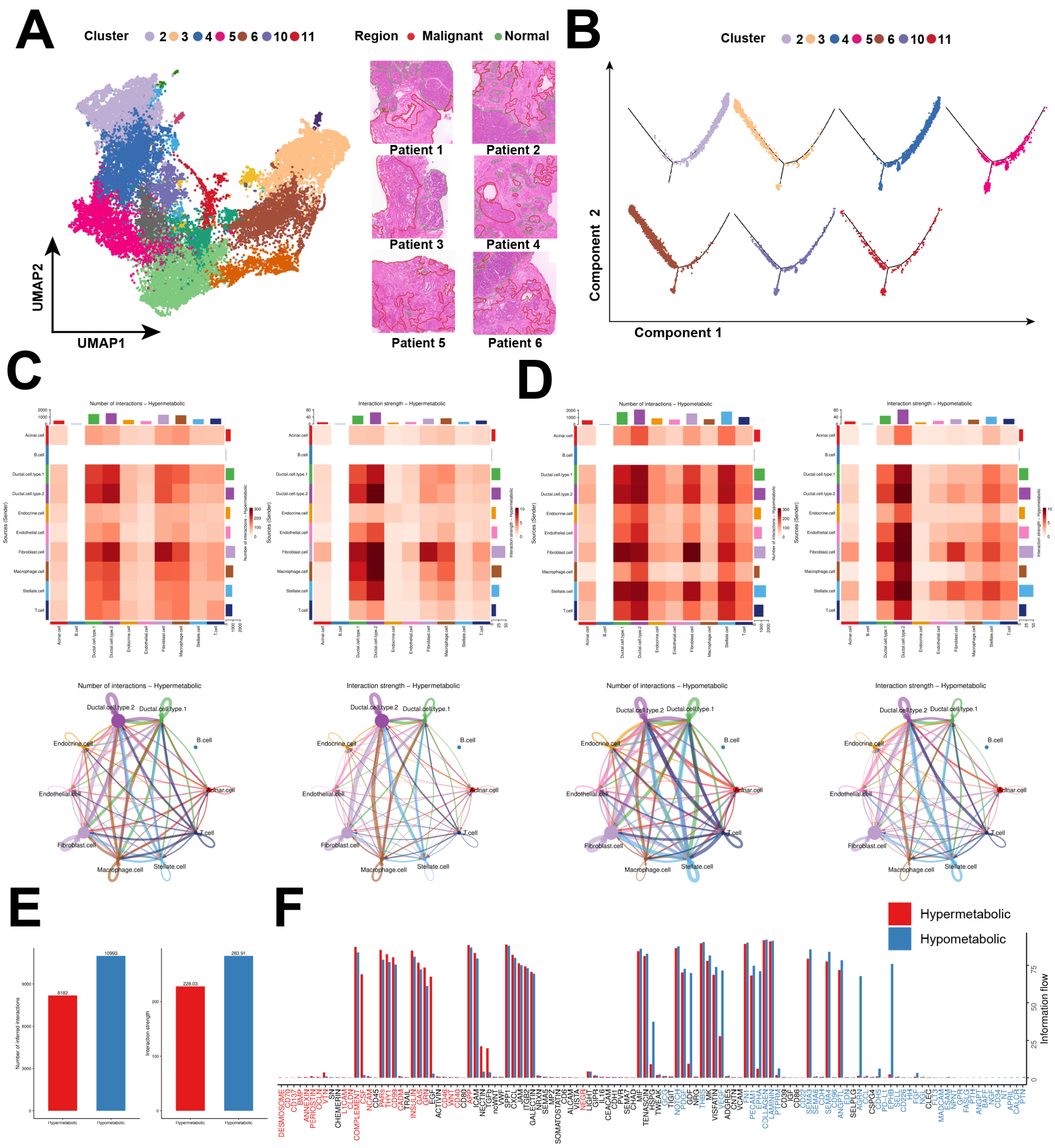

A: Schematic diagram of the recognition strategy of PDAC. Spots both located in UMAP plot and pathological regions were extracted for further pseudotime analysis. B: Trajectory plots showing the distribution of selected cells in the pseudotime trajectory split by cluster. Heat maps and network diagrams were used to display the number and strength of cell-cell interactions in hypermetabolic (C) and hypometabolic (D) regions. The bar graph shows the total number and strength of cell-cell interactions (E) and the information flow/interaction strength of each signaling pathway (F).

Figure S7 Differences in metabolic pathways between pathological regions

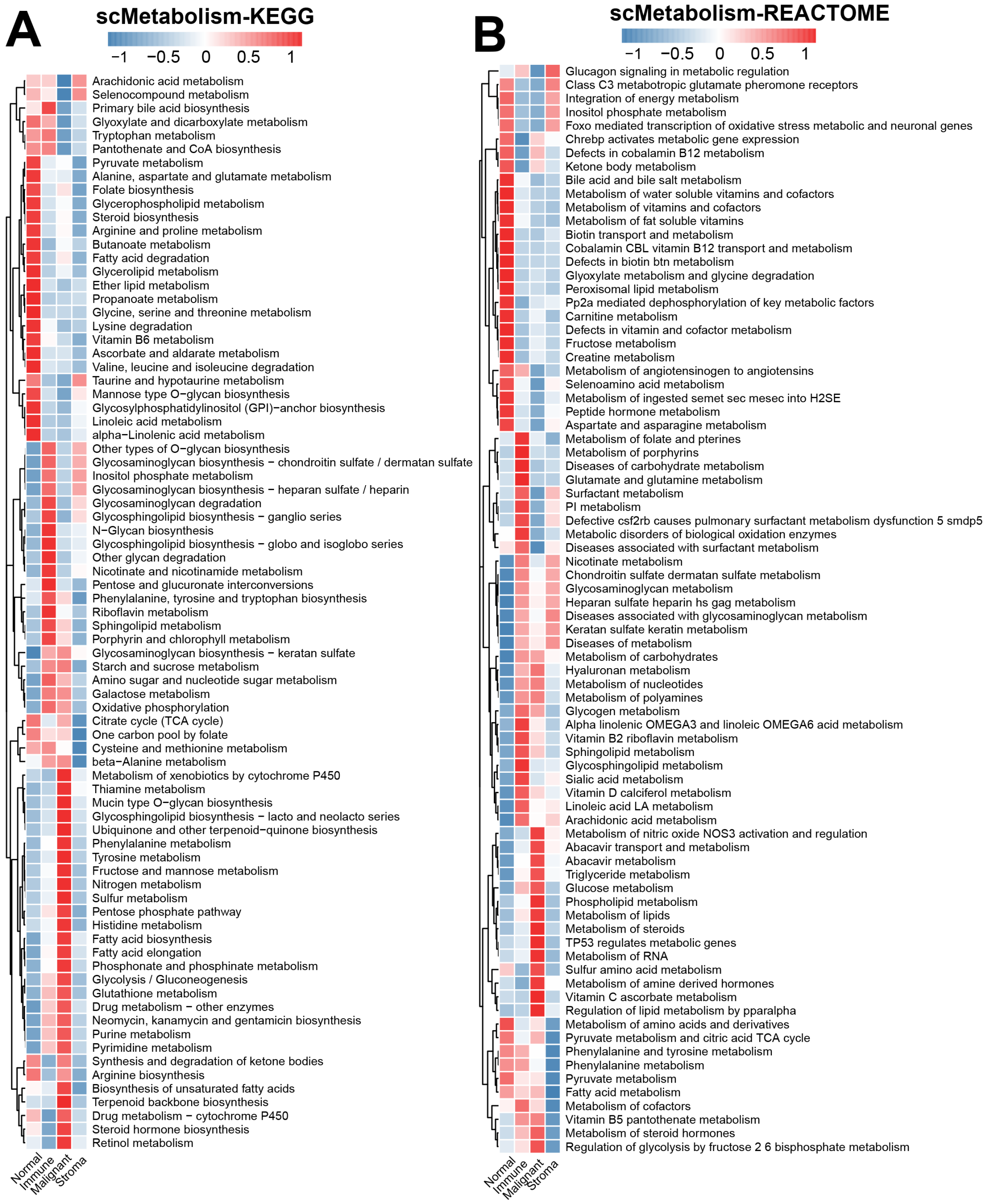

The enrichment of metabolism-related pathways among different pathological regions, including KEGG pathways (A) and REACTOME pathways (B), was analyzed by scMetabolism.

Figure S8 Spatial enrichment of representative pathways.

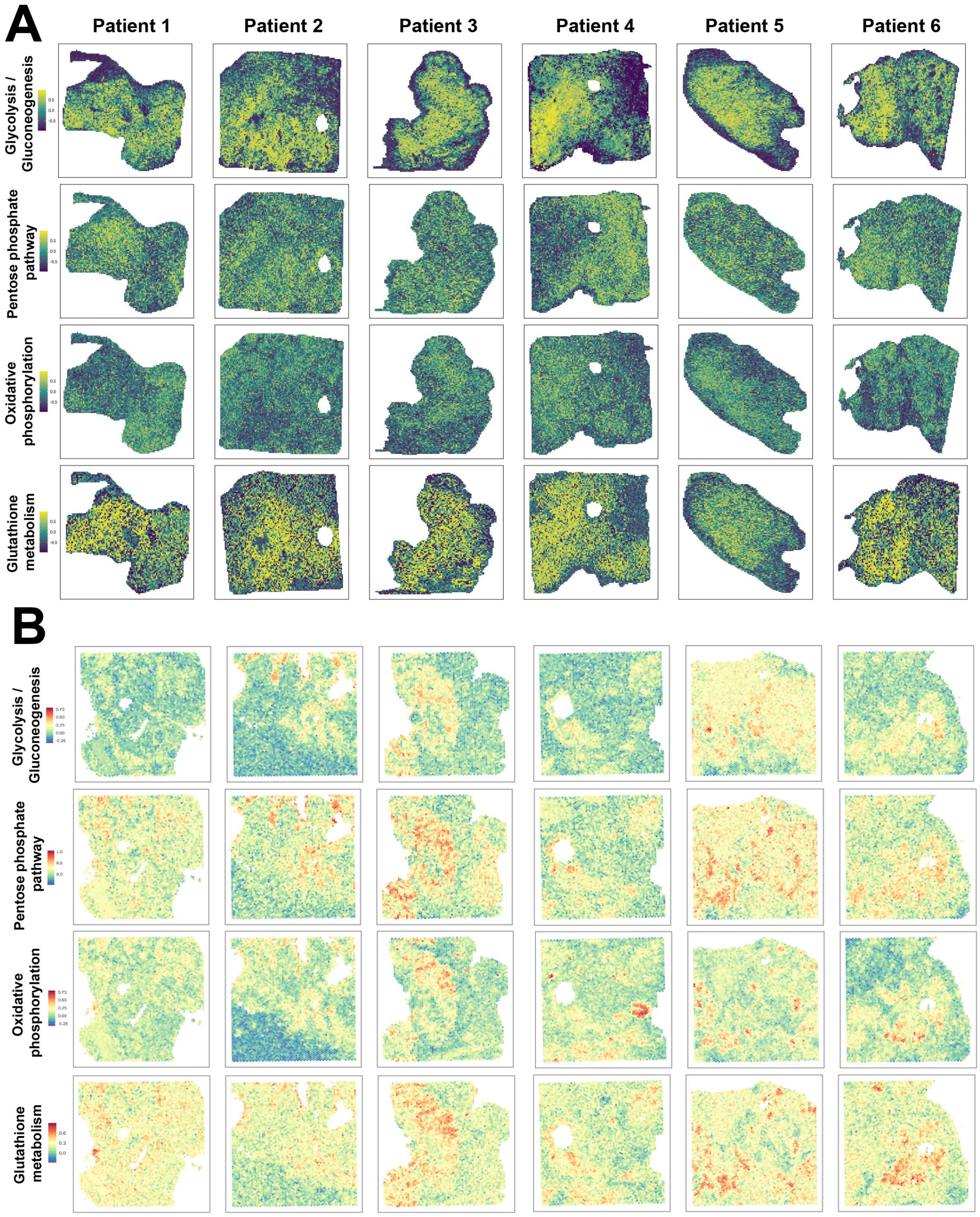

Visualize the enrichment of selected pathways through spatial metabolomics (A) and transcriptomics (B).

Figure S9 Expression and overall survival of ligand-receptor pairs in the TCGA-PAAD cohort (part 1).

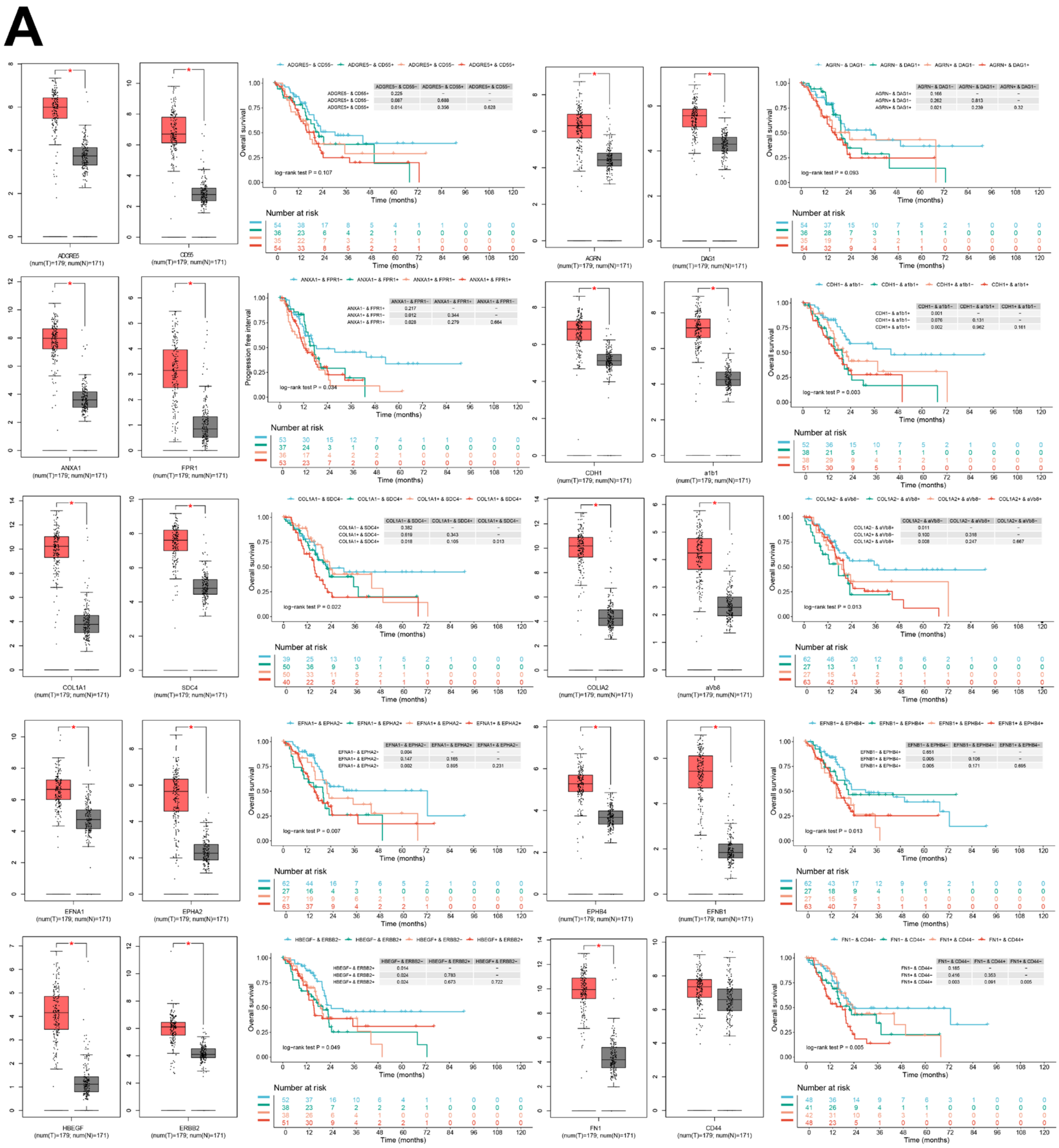

The clinical significances of ligand-receptor pairs in PAAD cohort of TCGA. The correlation between transcriptomic level (left, middle) and prognosis (right) were presented. Statistical analysis was performed by student's t test. P value < 0.05 was considered as statistical significance.

Figure S10 Expression and overall survival of ligand-receptor pairs in the TCGA-PAAD cohort (part 2).

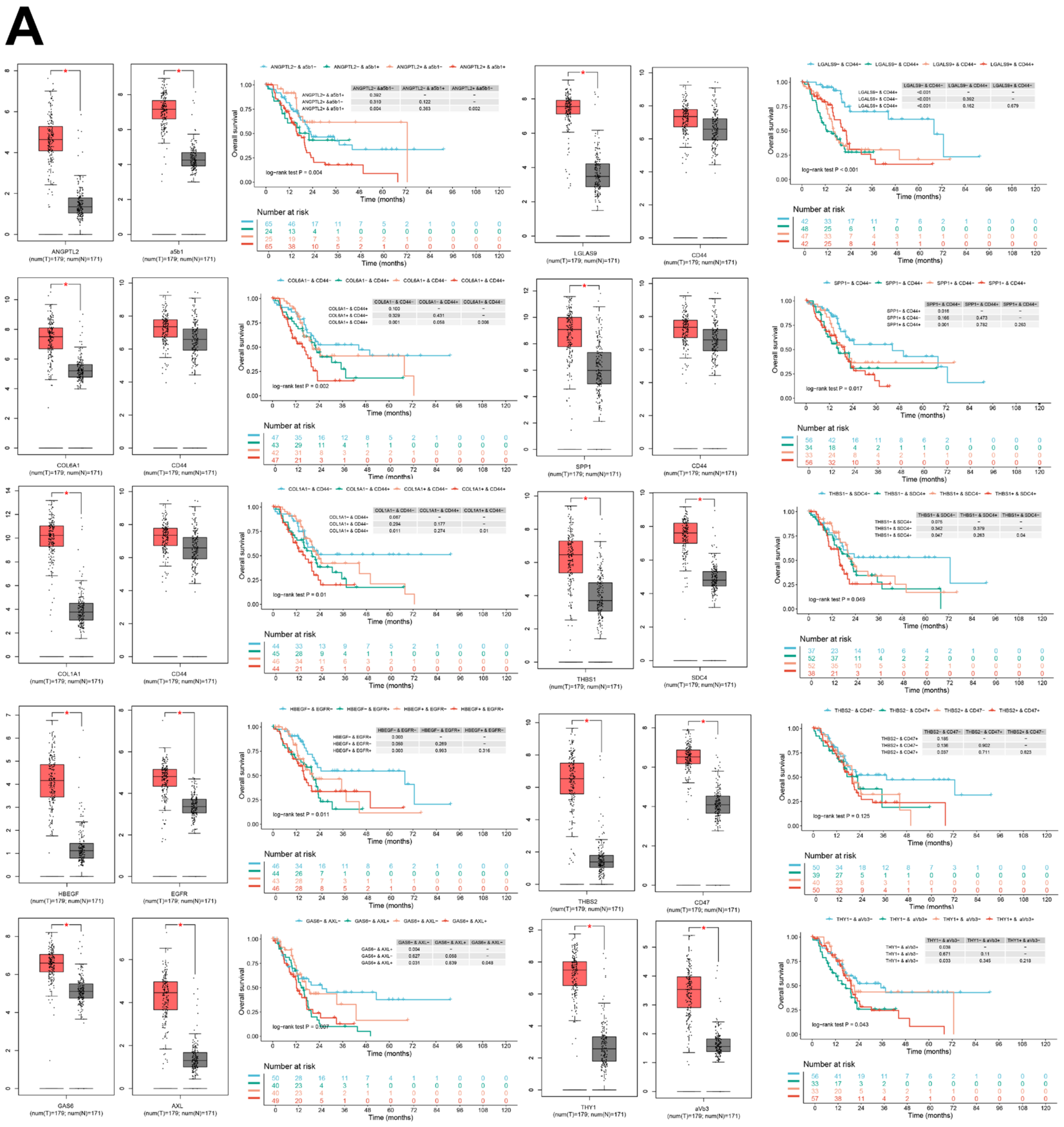

The clinical significances of ligand-receptor pairs in PAAD cohort of TCGA. The correlation between transcriptomic level (left, middle) and prognosis (right) were presented. Statistical analysis was performed by student’s t test. P value < 0.05 was considered as statistical significance.

Figure S11 ST feature plots exhibiting the expression level and spatial distribution of representative ligand–receptor pairs

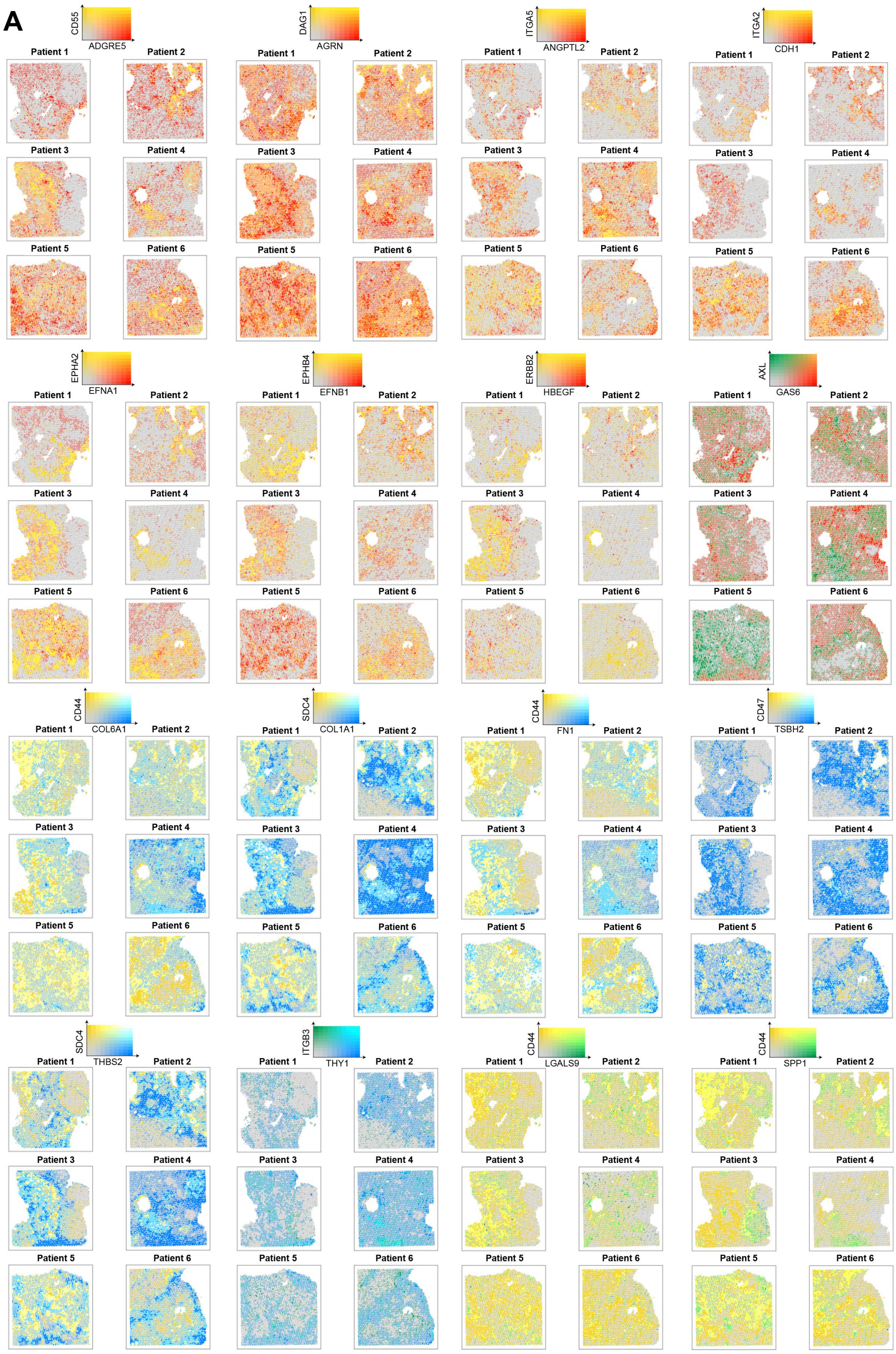

ST plot demonstrating the expression patterns of representative ligand-receptor pairs in 6 PDAC samples.

Figure S12 Regulon analysis in different metabolic regions by SCENIC

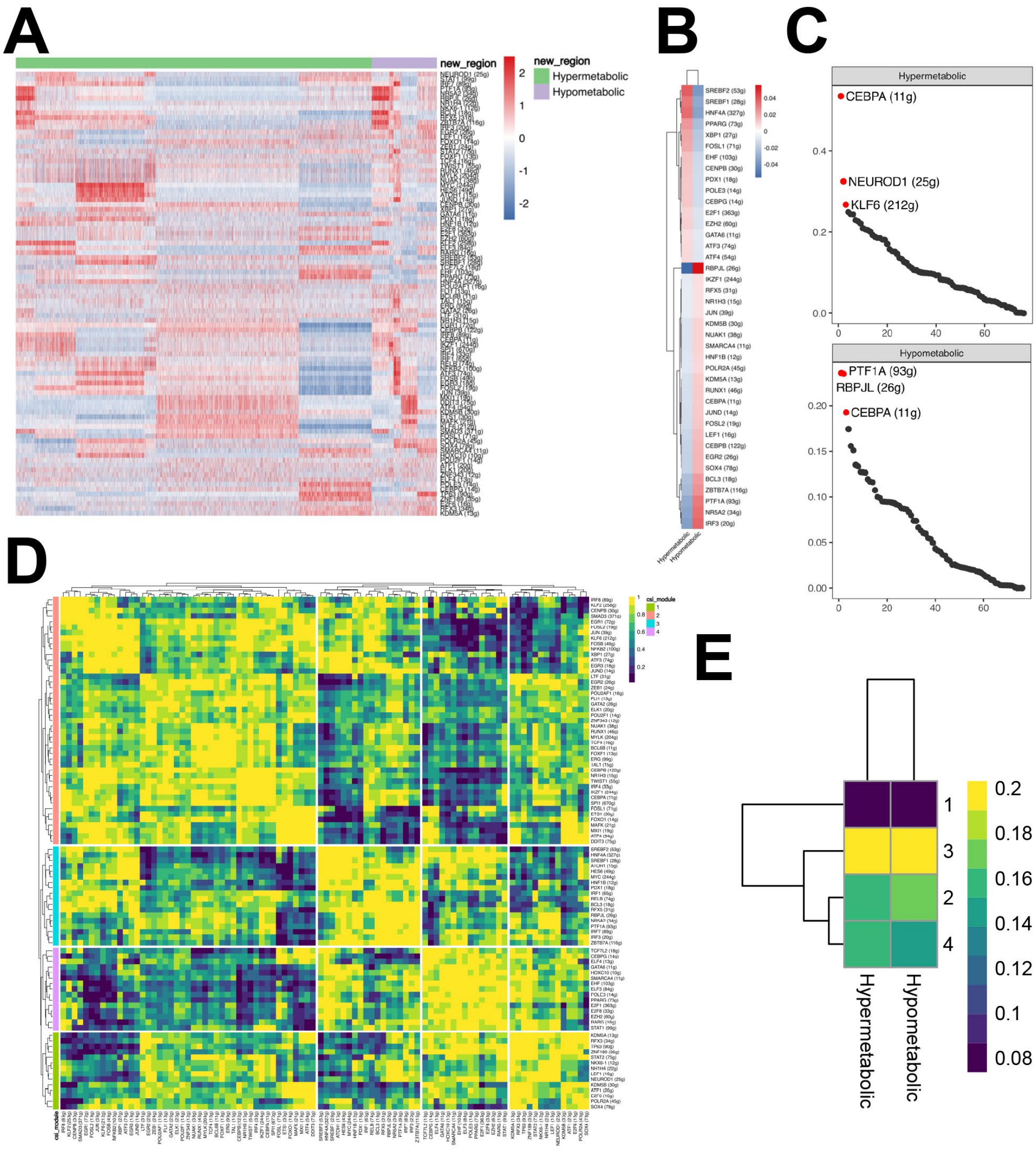

A: Heatmap of RAS activity of regulons in hypermetabolic and hypometabolic regions. B: Heatmap of RAS activity of regulons in duct cell type 2. C: Regulon-specific ranking map. D: CSI-associated clustering heatmap of Regulon modules. E: Activity heatmap of CSI-associated modules.

Figure S13 Spatial metabolomics characteristics of 6 PDAC samples.

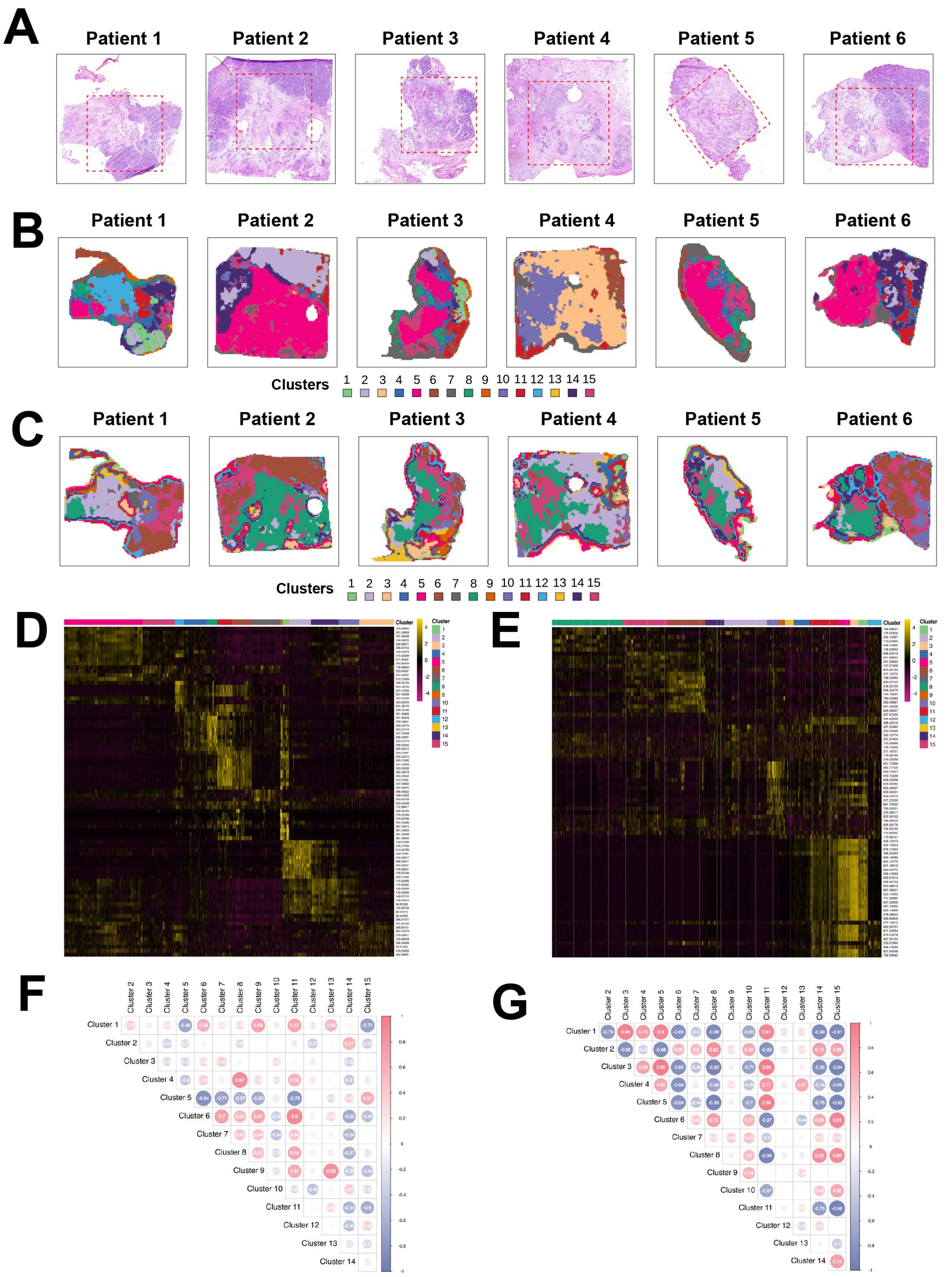

A: Due to the different detection areas, the correspondence between the SM area and the ST area is shown (ST is in the red line). B-C: Spatial shrunken centroids clustering (SCCC) of six samples, showing clustering diagrams through negative mode (B) and positive mode (C). D-E: Feature expression heat map shows the top 10 characteristic metabolites of each cluster above, in negative mode (D) and positive mode (E). F-G: Correlation analysis can measure the closeness of correlation between different clusters, in negative mode (F) and positive mode (G).

Figure S14 Spatial metabolomics features of different regions.

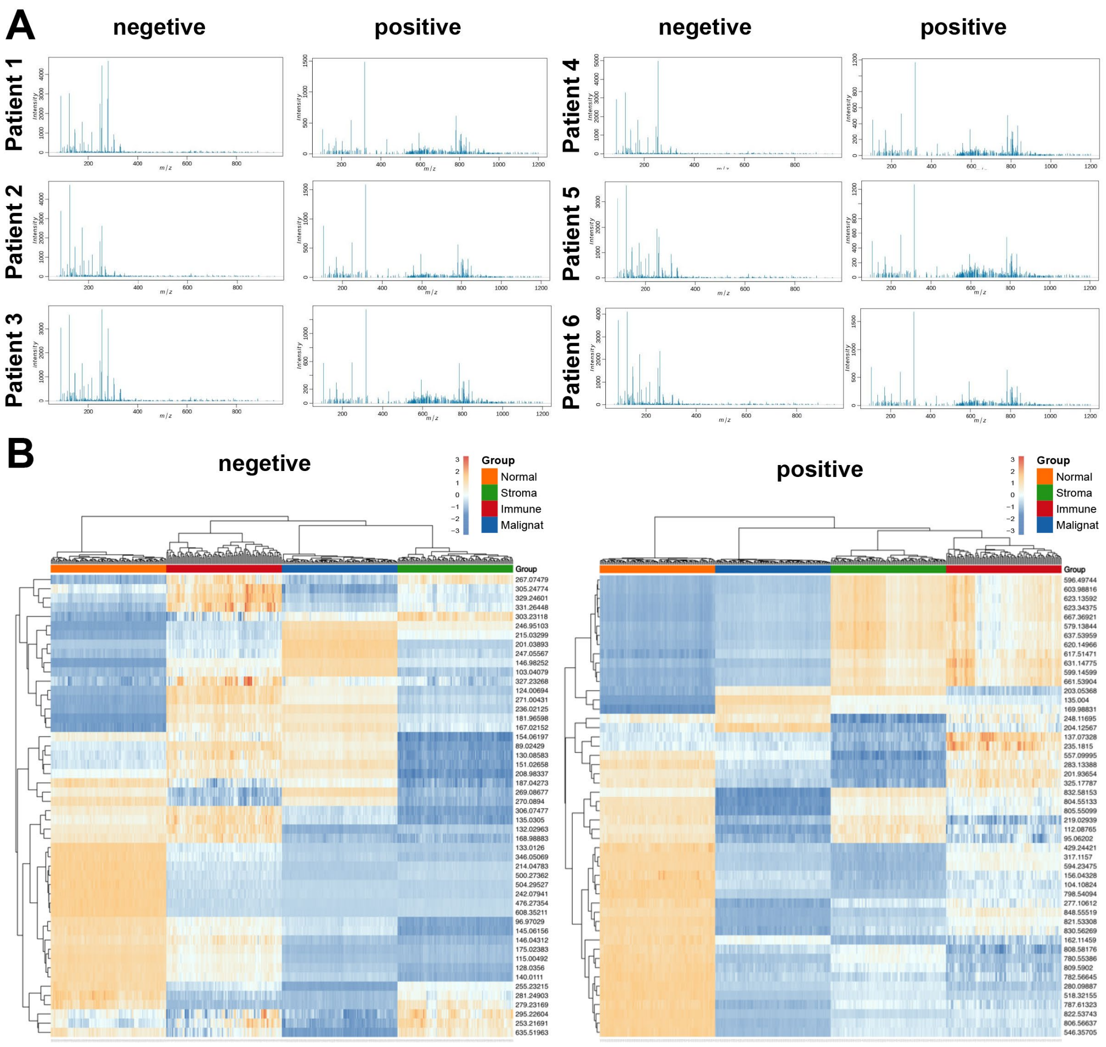

A: Mass spectrum images revealing the average abundance of mass spectrum in the 6 samples detected by AFADESI-MSI. B: Heatmap displaying the abundance of top 10 m/z metabolites in each histopathological region of 6 samples

Figure S15 Visualization of the abundance and distribution of key metabolites associated with carbohydrate metabolism.

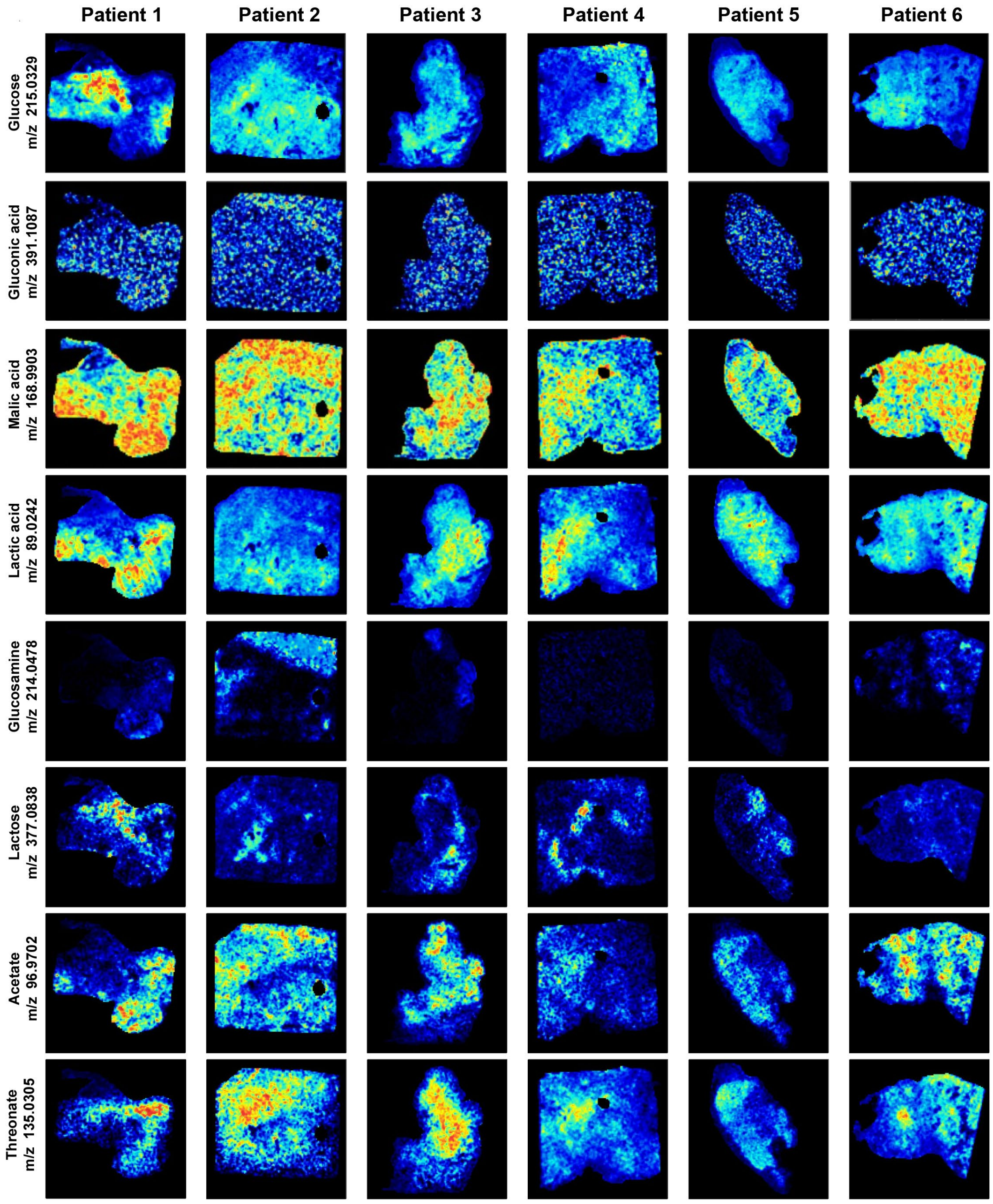

Figure S16 Visualization of the abundance and distribution of key metabolites associated with amino acid metabolism (part 1).

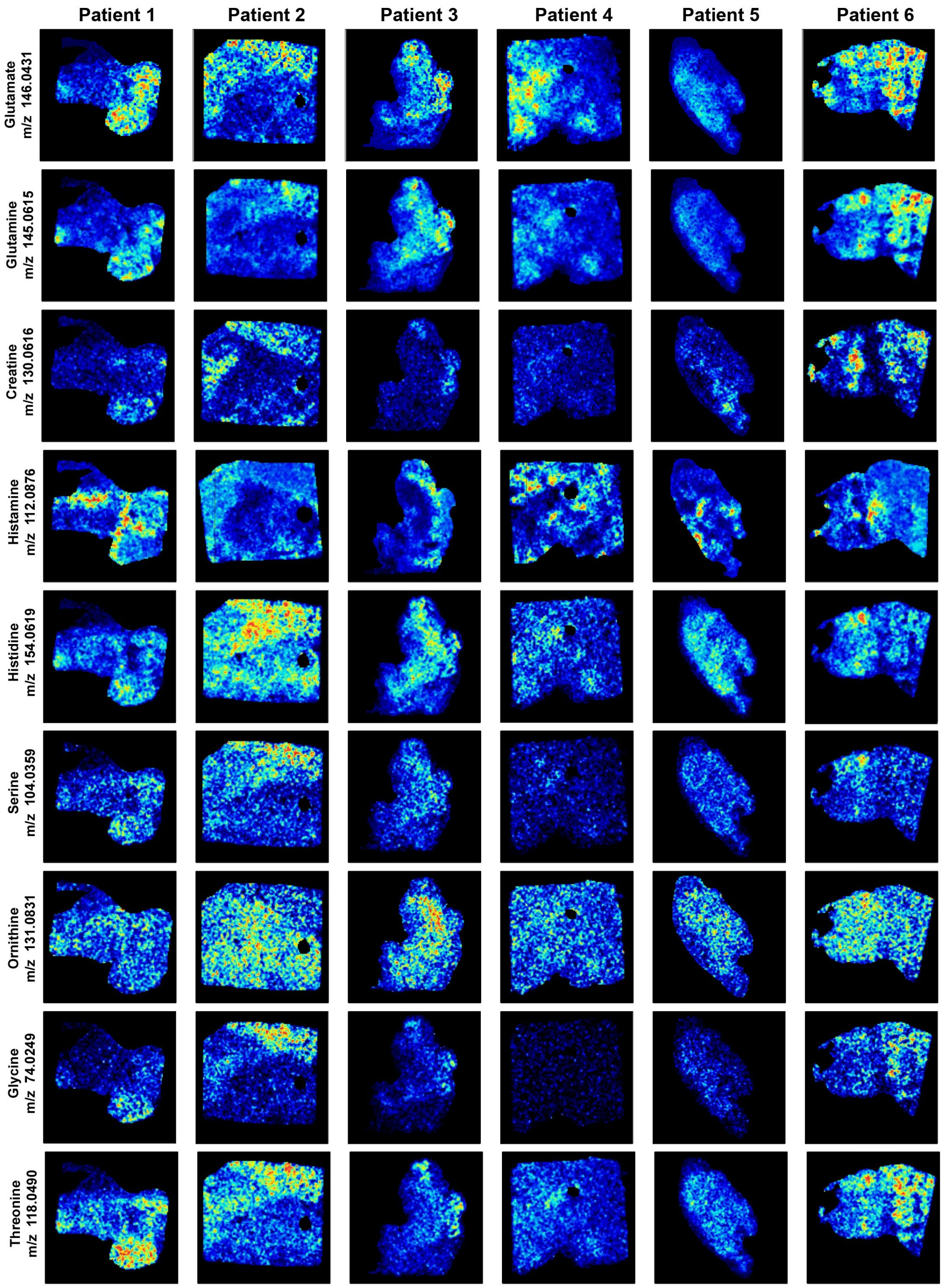

Figure S17 Visualization of the abundance and distribution of key metabolites associated with amino acid metabolism (part 2).

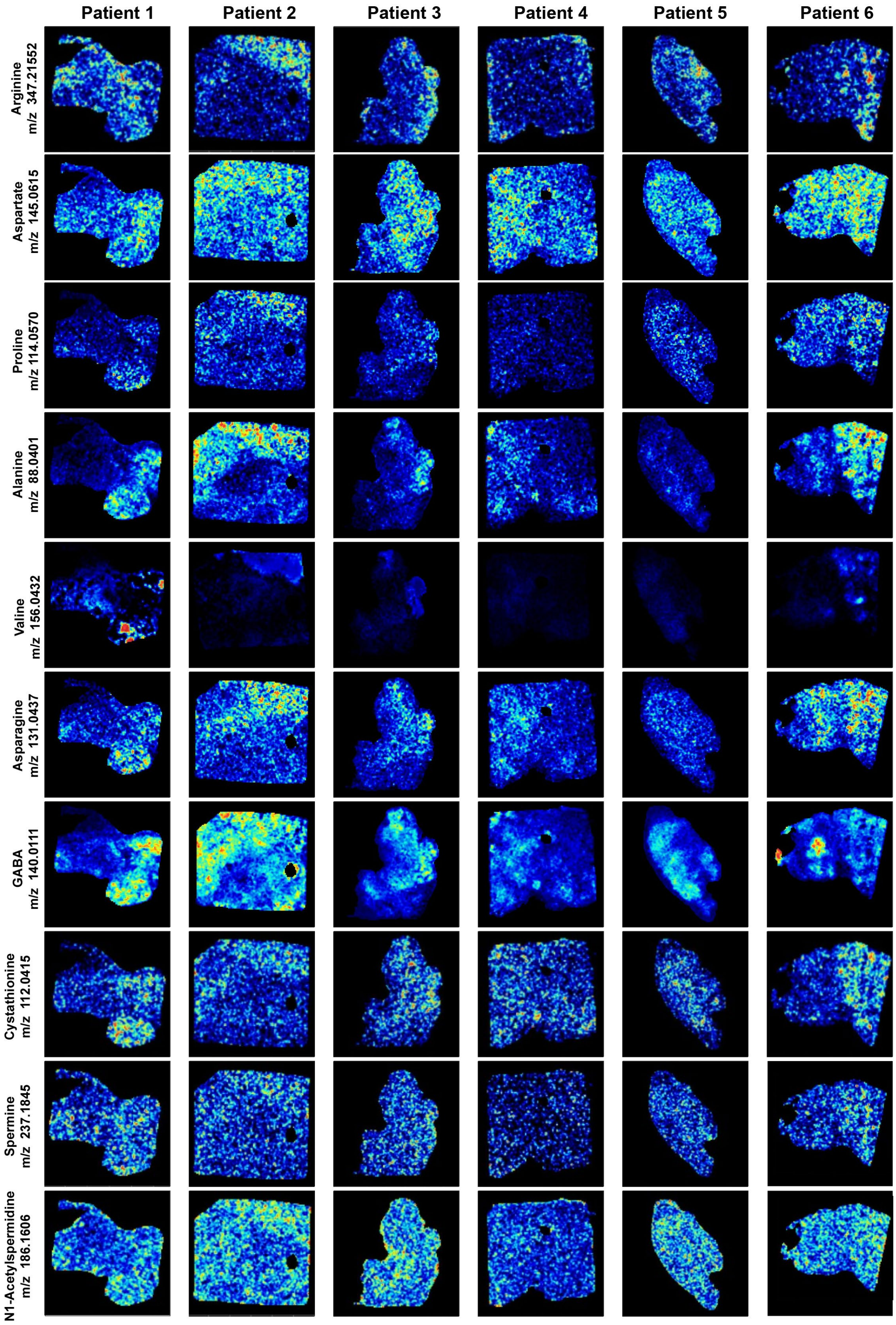

Figure S18 Visualization of the abundance and distribution of key metabolites associated with lipid metabolism (part 1).

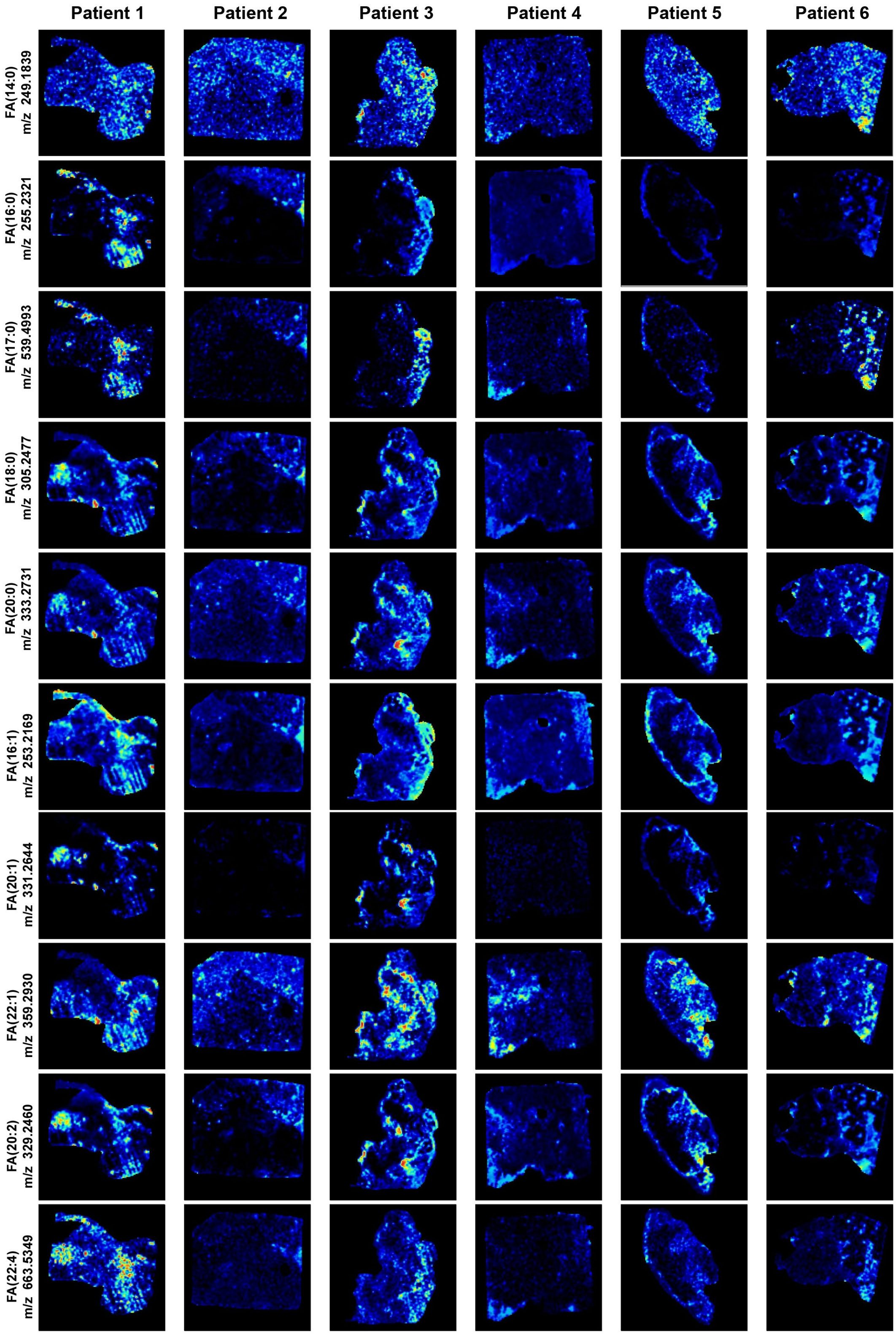

Figure S19 Visualization of the abundance and distribution of key metabolites associated with lipid metabolism (part 2).

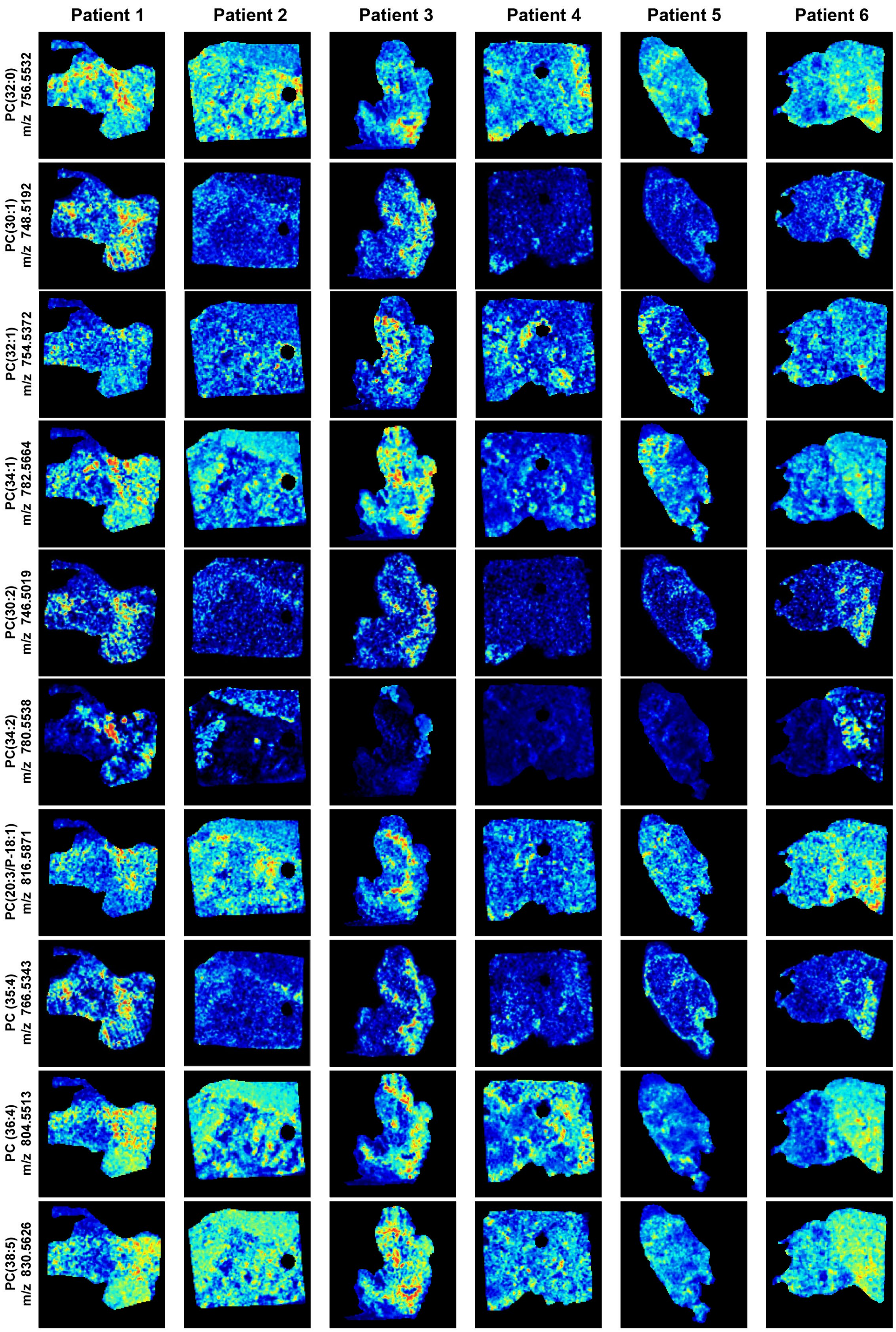

Figure S20 Visualization of the abundance and distribution of key metabolites associated with lipid metabolism (part 3).

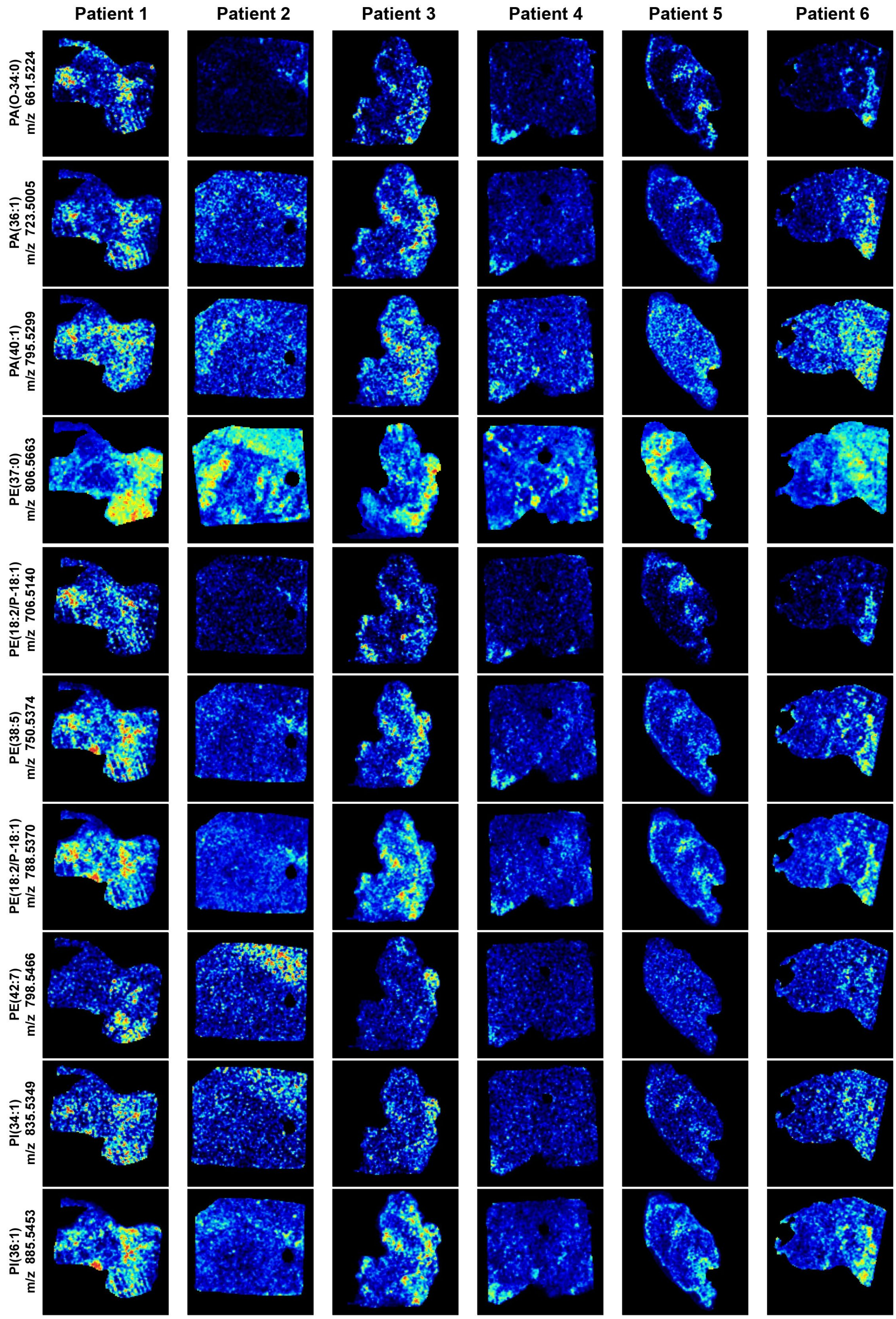

Figure S21 Visualization of the abundance and distribution of key metabolites associated with lipid metabolism (part 4).

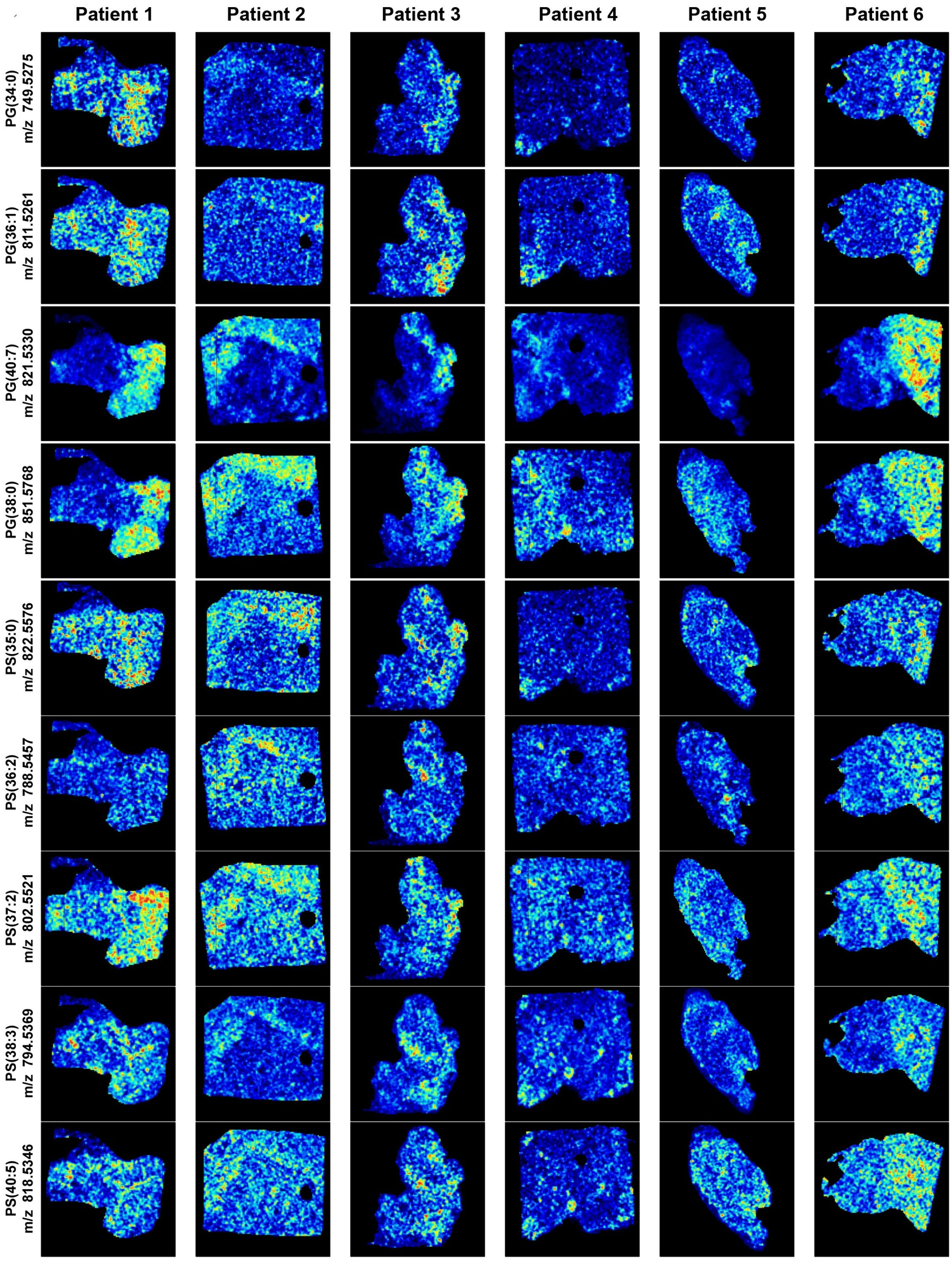

Figure S22 Visualization of the abundance and distribution of key metabolites associated with lipid metabolism (part 5).

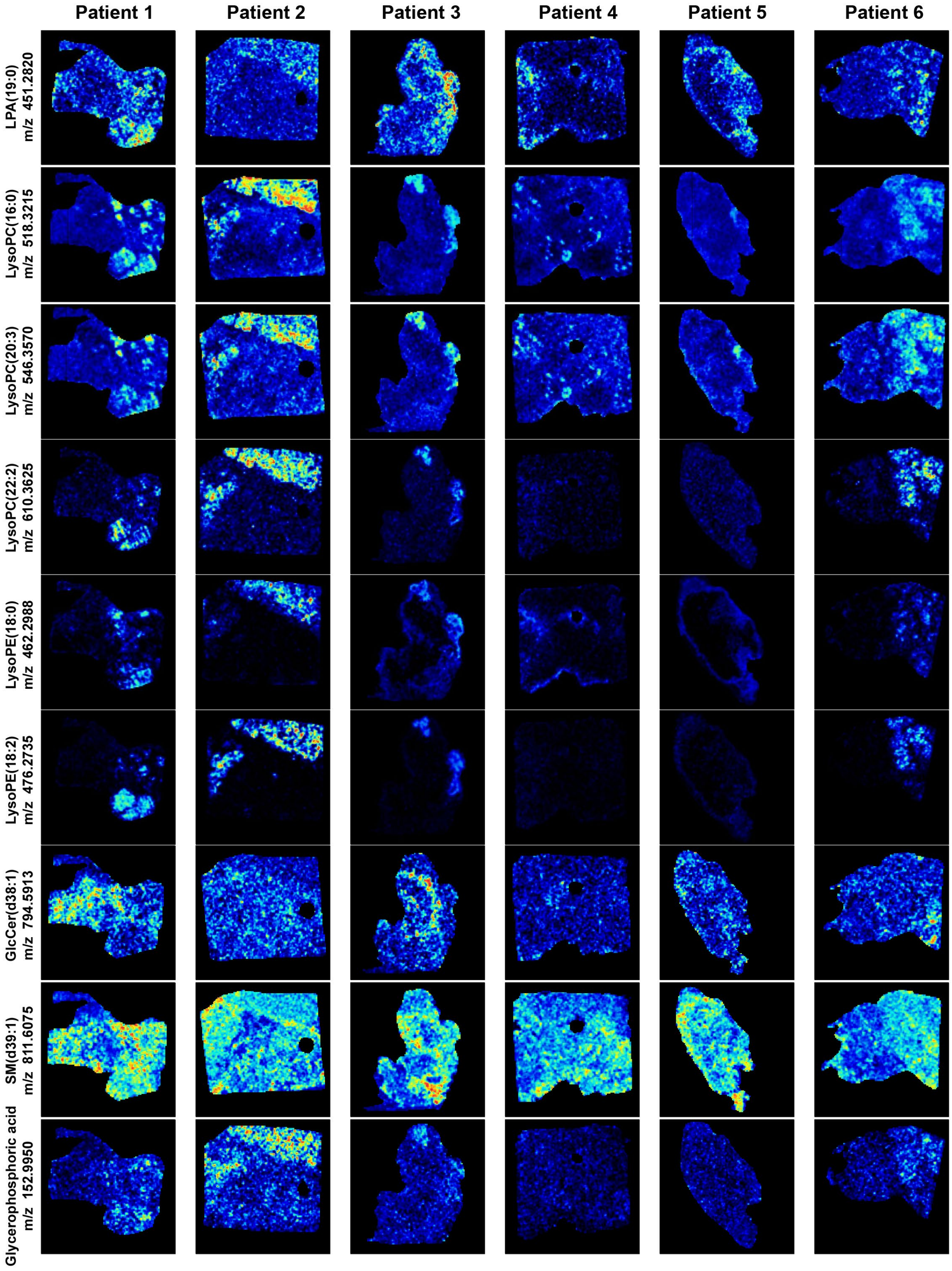

Figure S23 Visualization of the abundance and distribution of key metabolites associated with nucleotide metabolism.

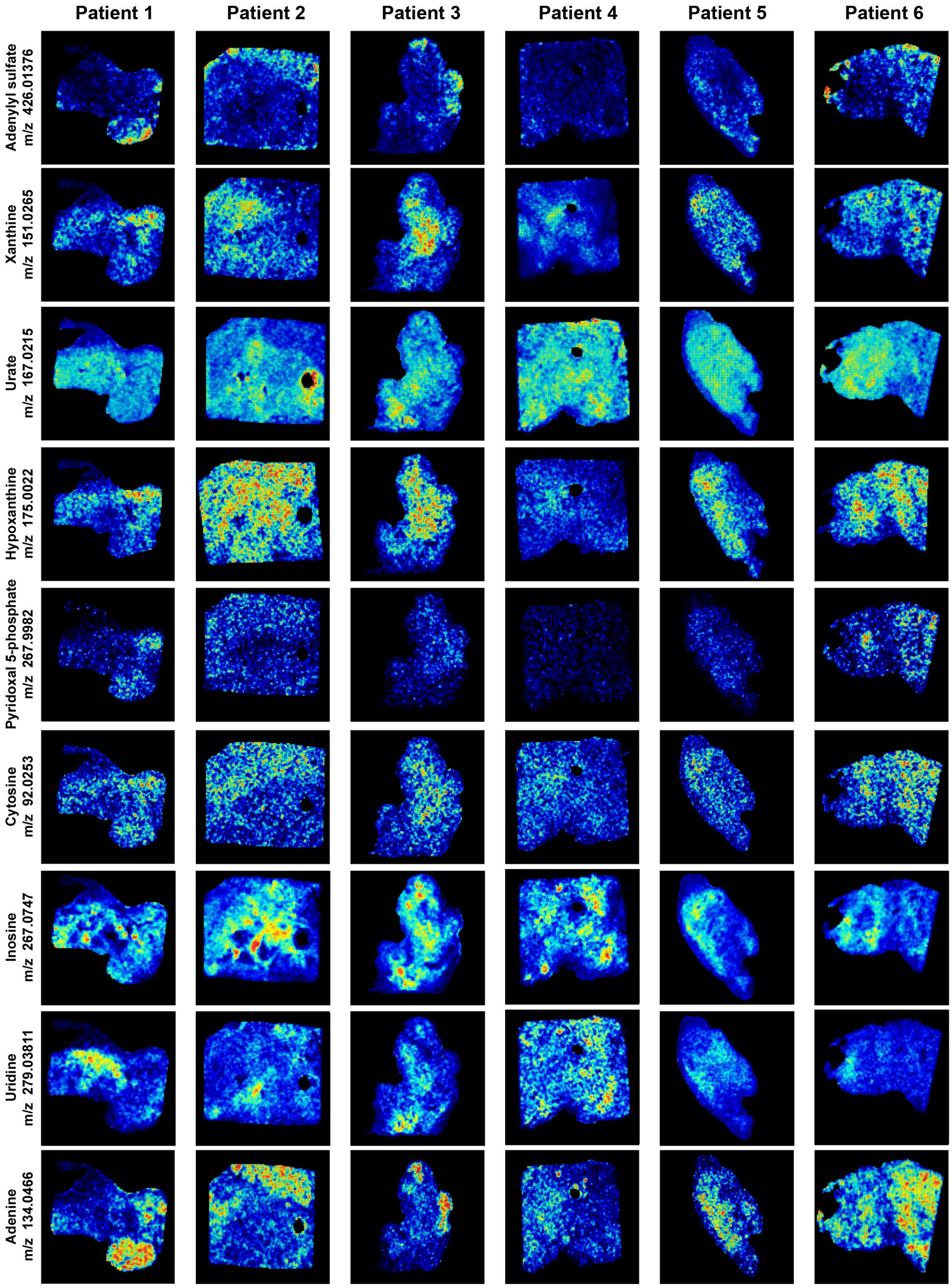

Figure S24 Visualization of the abundance and distribution of key metabolites associated with others metabolism.

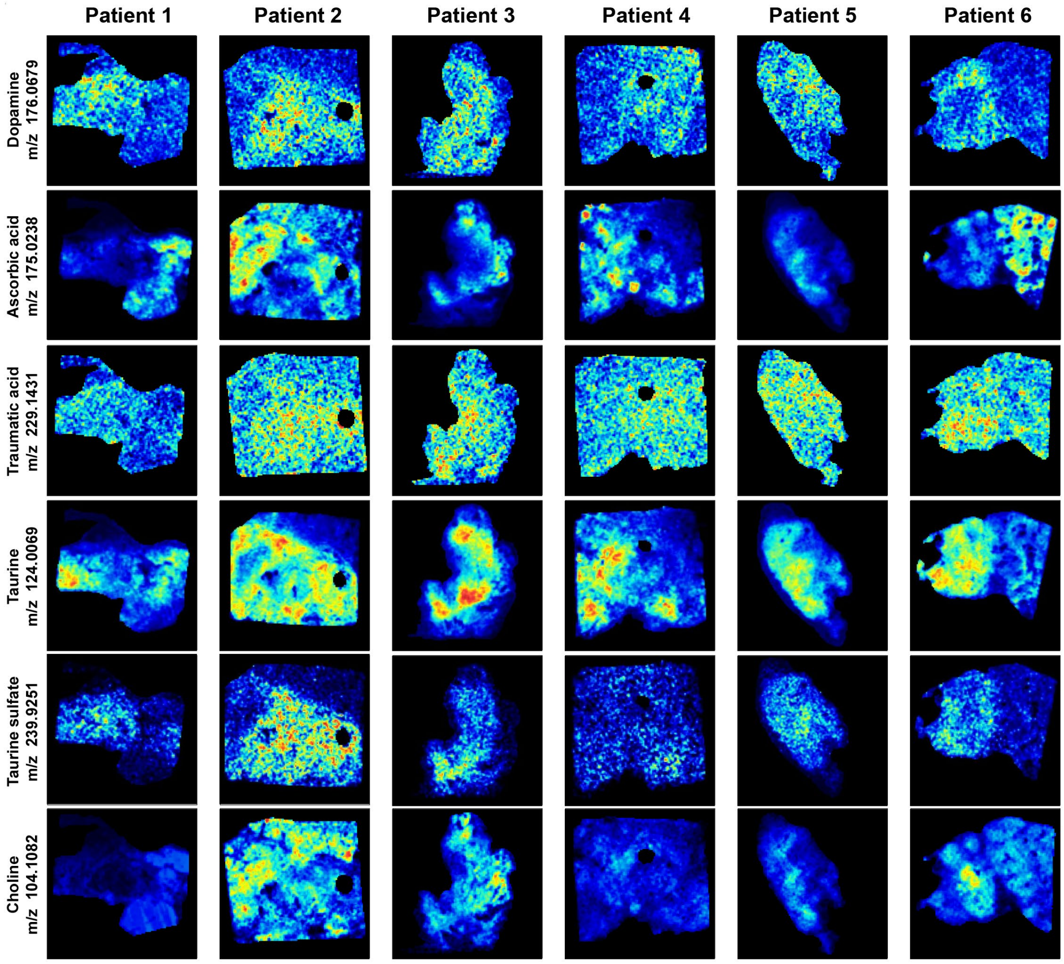

Figure S25 Enrichment analysis between malignant and normal regions.

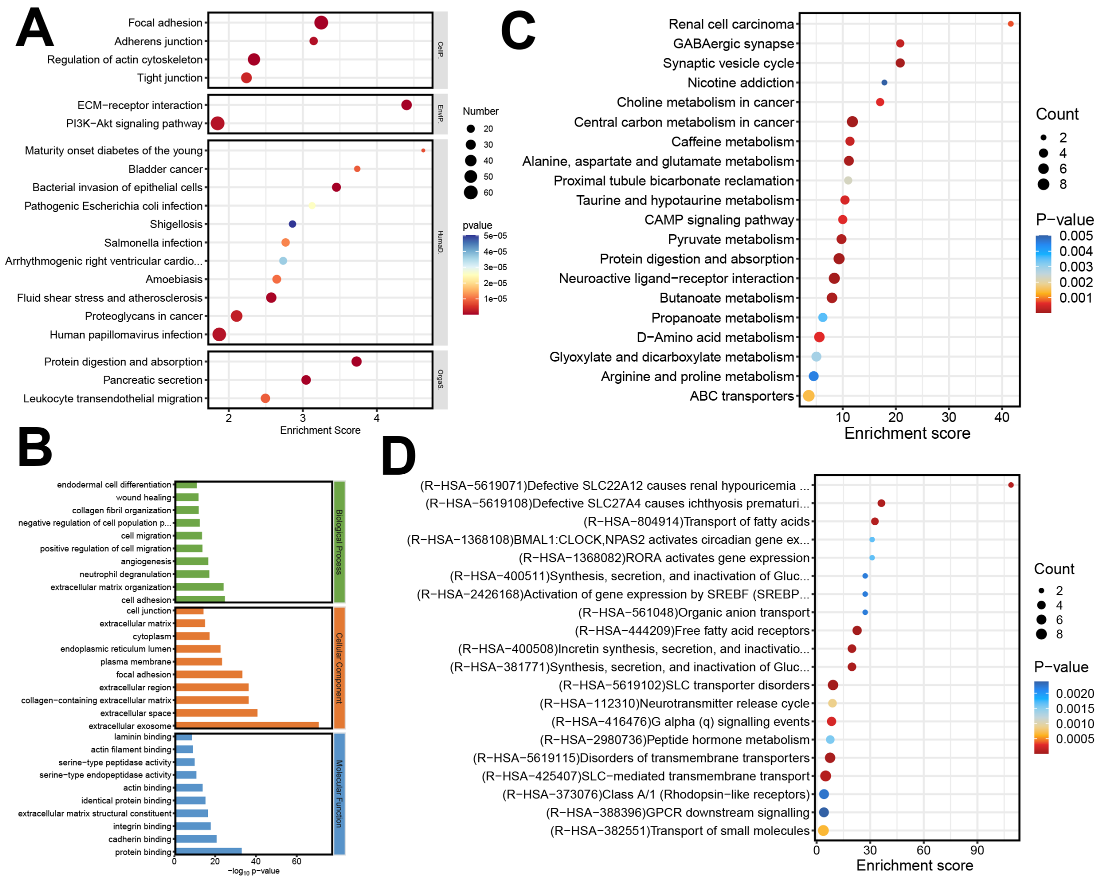

A: KEGG pathway enrichment of spatial transcriptomics. B: KEGG pathway enrichment of spatial metabolomics. C: GO pathway enrichment of spatial transcriptomics.

D: REACTOME pathway enrichment of spatial transcriptomics.

Figure S26 Enrichment analysis between malignant and stroma regions.

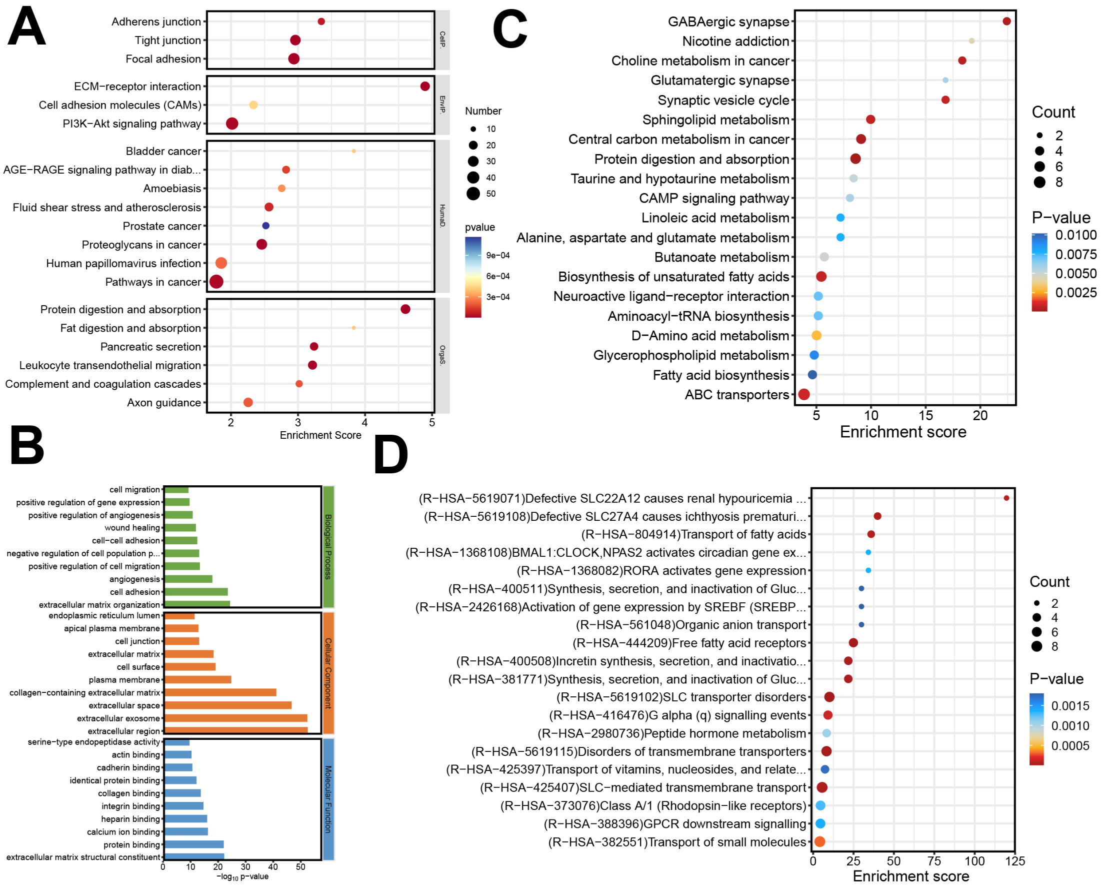

A: KEGG pathway enrichment of spatial transcriptomics. B: KEGG pathway enrichment of spatial metabolomics. C: GO pathway enrichment of spatial transcriptomics.

D: REACTOME pathway enrichment of spatial transcriptomics.

Figure S27 Enrichment analysis between immune and stroma regions.

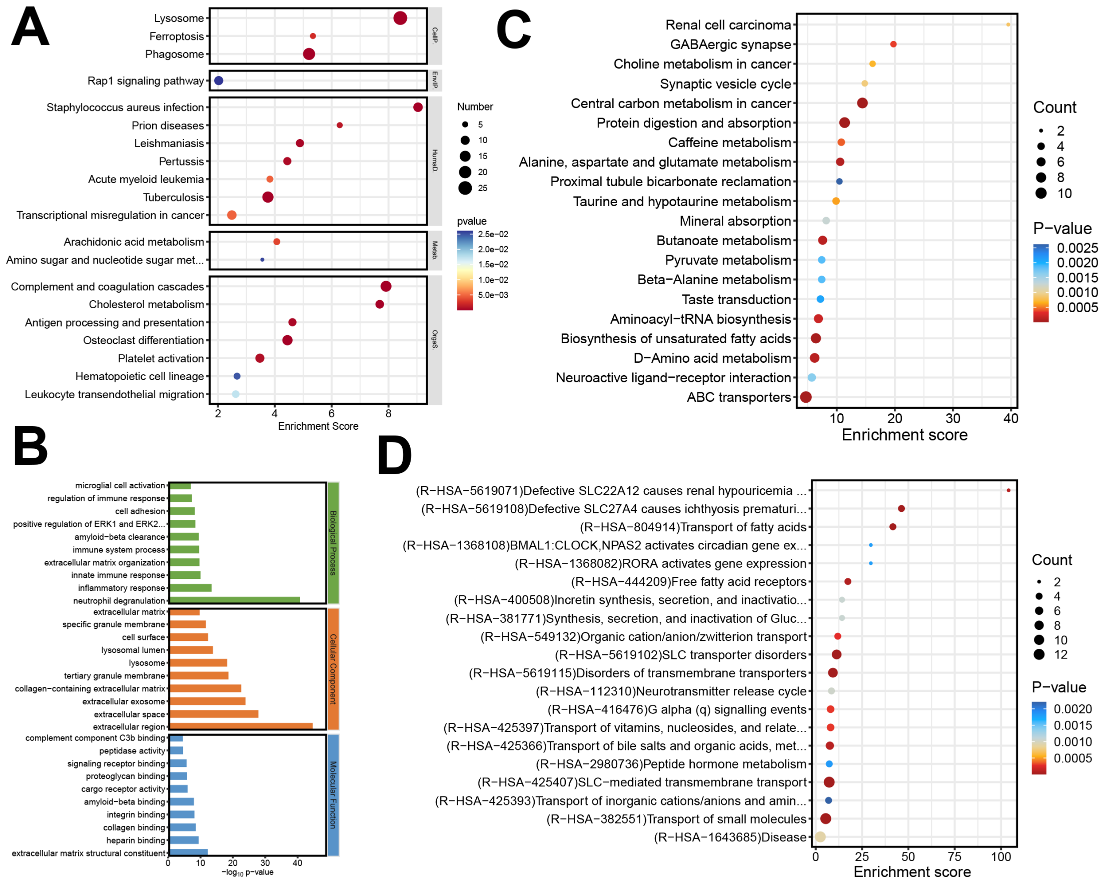

A: KEGG pathway enrichment of spatial transcriptomics. B: KEGG pathway enrichment of spatial metabolomics. C: GO pathway enrichment of spatial transcriptomics.
D: REACTOME pathway enrichment of spatial transcriptomics.

Figure S28 Enrichment analysis between neoadjuvant treatment and no treatment in malignant regions.

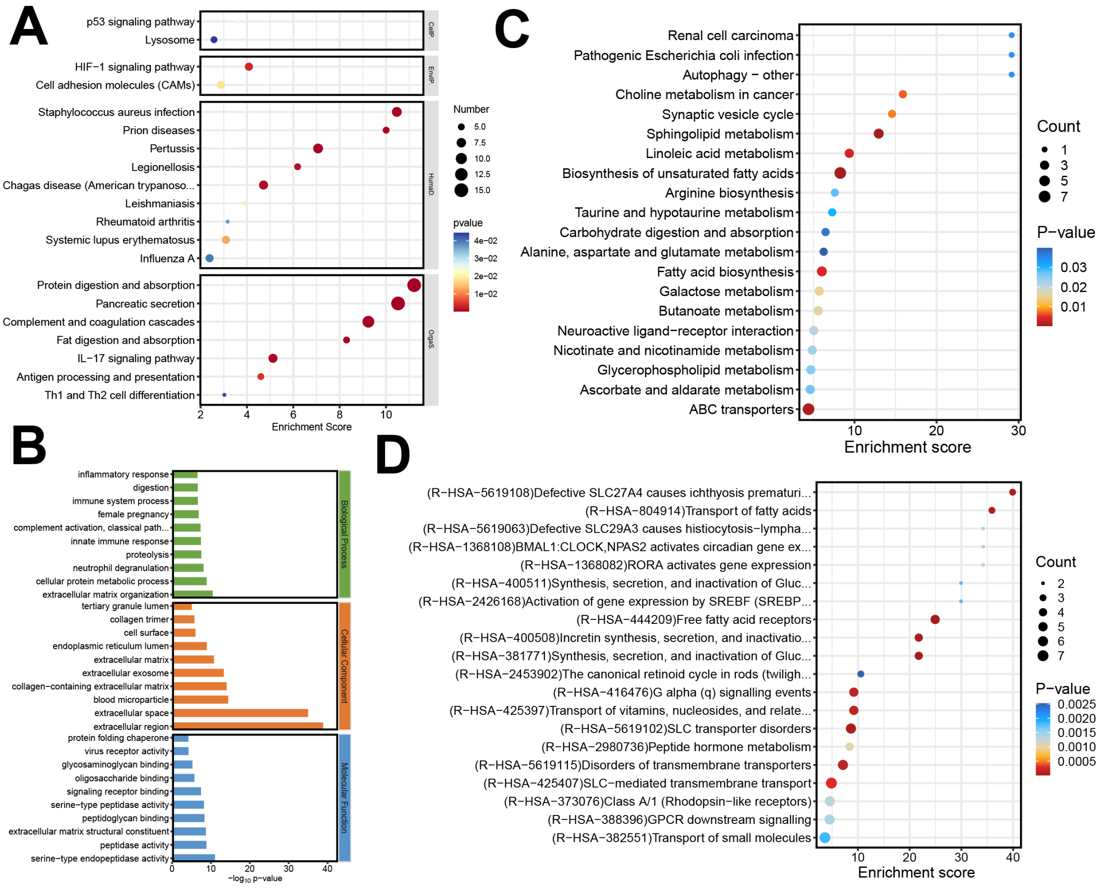

A: KEGG pathway enrichment of spatial transcriptomics. B: KEGG pathway enrichment of spatial metabolomics. C: GO pathway enrichment of spatial transcriptomics.

D: REACTOME pathway enrichment of spatial transcriptomics.

Figure S29 Enrichment analysis between neoadjuvant treatment and no treatment in stroma regions.

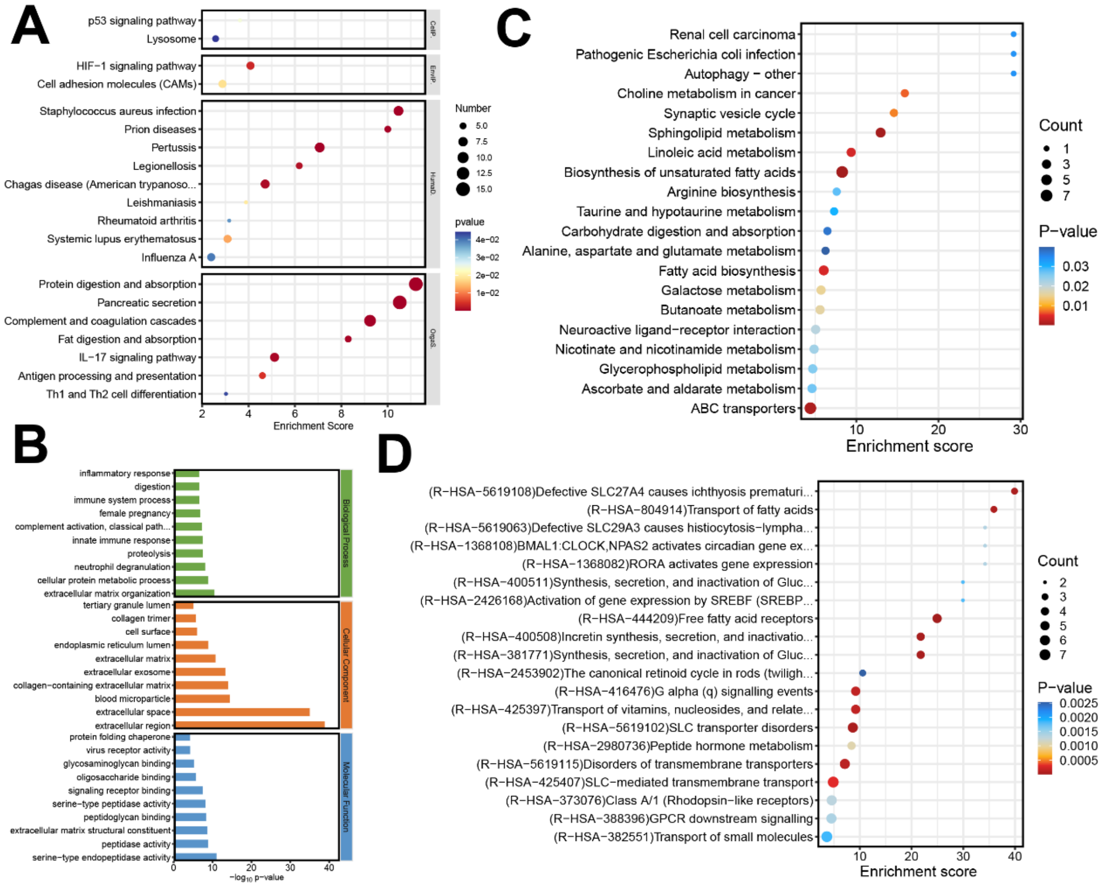

A: KEGG pathway enrichment of spatial transcriptomics. B: KEGG pathway enrichment of spatial metabolomics. C: GO pathway enrichment of spatial transcriptomics.
D: REACTOME pathway enrichment of spatial transcriptomics.

Table S1 Characteristics of patients included in this study

Patient ID	Gender	Age	Ancestry	Race	Tumor Position	Tumor Size	TNM Stage	Clinical Stage	Neoadjuvant Treatment
1	Male	71	East Asian	Asian	Head	5.0	T3N0M0	IIA	No
2	Female	34	East Asian	Asian	Body/tail	4.0	T2N0M0	IB	Yes
3	Female	72	East Asian	Asian	Body/tail	2.0	T2N1M0	IIB	No
4	Male	50	East Asian	Asian	Body/tail	0.6	T1N0M0	IA	No
5	Female	70	East Asian	Asian	Body/tail	2.0	T2N1M0	IIB	Yes
6	Male	41	East Asian	Asian	Head	2.0	T2N1M0	IIB	No

Table S4 Representative differentially expressed genes in amino acid and lipid metabolic networks across pathological subregions of spatial transcriptomics

Gene	p-value	log2FoldChange	pct.1	pct.2	q-value
ELOVL1	0.00E+00	1.2770	0.9700	0.5800	0.00E+00
ASL	0.00E+00	0.5810	0.9150	0.7310	0.00E+00
CD36	0.00E+00	-0.3666	0.0530	0.2890	0.00E+00
CPA2	0.00E+00	-3.7306	0.1970	0.8010	0.00E+00
DGAT1	0.00E+00	0.6873	0.8050	0.4710	0.00E+00
SCD	0.00E+00	1.3215	0.7040	0.2500	0.00E+00
PLPP4	0.00E+00	0.4552	0.3080	0.0630	0.00E+00
PLA2G1B	0.00E+00	-4.0410	0.2090	0.7850	0.00E+00
LPCAT4	0.00E+00	1.6309	0.9160	0.3810	0.00E+00
KRT19	0.00E+00	2.3362	0.9660	0.2960	0.00E+00
FASN	0.00E+00	1.0571	0.8830	0.5840	0.00E+00
PLPP2	0.00E+00	0.6153	0.7380	0.4520	0.00E+00
SMS	0.00E+00	0.8560	0.8400	0.4850	0.00E+00
SLC27A4	2.38E-285	0.4970	0.6540	0.4220	4.02E-281
AGPAT2	2.52E-223	0.4913	0.8800	0.7250	4.25E-219
ASS1	1.15E-222	0.7197	0.5140	0.3180	1.94E-218
GPT	1.80E-201	-0.2951	0.1420	0.3350	3.03E-197
GOT2	2.22E-149	0.3602	0.5370	0.3630	3.75E-145
CHKA	4.70E-138	-0.3291	0.8220	0.8770	7.92E-134
ACACA	1.89E-127	0.3142	0.4320	0.2670	3.18E-123
PYCR1	1.04E-122	0.3309	0.5040	0.3460	1.75E-118
ACCS	1.90E-110	-0.2746	0.3670	0.5250	3.19E-106
LPCAT1	4.68E-104	0.2797	0.7150	0.5560	7.89E-100
CHPT1	8.15E-101	0.2863	0.3580	0.2160	1.37E-96
ACSL3	2.03E-83	0.2519	0.3690	0.2390	3.42E-79
CDS2	1.70E-76	-0.2612	0.4820	0.5830	2.86E-72
ELOVL1	0.00E+00	0.7621	0.9700	0.8630	0.00E+00
ASL	0.00E+00	0.5939	0.9150	0.7320	0.00E+00

SFRP1	0.00E+00	-0.8664	0.0960	0.3310	0.00E+00
AGPAT2	0.00E+00	0.7423	0.8800	0.6250	0.00E+00
SCD	0.00E+00	1.1400	0.7040	0.3380	0.00E+00
LPCAT4	0.00E+00	1.2412	0.9160	0.5900	0.00E+00
KRT19	0.00E+00	1.7099	0.9660	0.5710	0.00E+00
PLPP2	0.00E+00	0.8622	0.7380	0.3200	0.00E+00
SMS	0.00E+00	0.6091	0.8400	0.6420	0.00E+00
ASS1	4.76E-308	0.7313	0.5140	0.2690	8.02E-304
FASN	1.61E-265	0.7048	0.8830	0.7990	2.71E-261
CD3E	7.02E-243	-0.4051	0.0780	0.2540	1.18E-238
DGAT1	5.58E-239	0.3999	0.8050	0.6230	9.40E-235
SAT1	6.17E-153	0.3357	0.9980	0.9870	1.04E-148
PYCR1	1.21E-152	0.3055	0.5040	0.3090	2.04E-148
GOT2	8.45E-147	0.3132	0.5370	0.3530	1.42E-142
SLC27A4	3.91E-116	0.2859	0.6540	0.5210	6.58E-112
ACACA	6.75E-116	0.2585	0.4320	0.2700	1.14E-111
FADS2	7.93E-97	-0.2750	0.3850	0.5290	1.34E-92
CPA2	1.47E-74	-0.4959	0.1970	0.3050	2.47E-70
PLA2G1B	3.88E-61	-0.4722	0.2090	0.3060	6.55E-57
HS3ST2	0.00E+00	-0.8212	0.1070	0.5240	0.00E+00
SAT1	2.16E-175	-0.6383	0.9870	1.0000	3.64E-171
GLUL	4.95E-128	-0.7867	0.9760	0.9730	8.34E-124
FABP3	1.08E-125	-0.4803	0.1200	0.3800	1.81E-121
PLD4	6.47E-54	-0.4913	0.2660	0.4680	1.09E-49
SCD	2.12E-51	-0.3581	0.3380	0.5470	3.57E-47
SMS	3.65E-46	-0.3375	0.6420	0.7760	6.15E-42
SFRP1	2.41E-34	0.7337	0.3310	0.1800	4.06E-30
ELOVL1	0.00E+00	-0.5149	0.5800	0.8630	0.00E+00
CPA2	0.00E+00	3.2347	0.8010	0.3050	0.00E+00
GPT	0.00E+00	0.4272	0.3350	0.0550	0.00E+00
PLA2G1B	0.00E+00	3.5688	0.7850	0.3060	0.00E+00
CHKA	8.09E-294	0.5050	0.8770	0.7330	1.36E-289
KRT19	1.36E-250	-0.6263	0.2960	0.5710	2.29E-246
GLUL	1.74E-185	-0.4416	0.9270	0.9760	2.93E-181
PLPP4	1.24E-179	-0.3436	0.0630	0.2340	2.08E-175
LPCAT4	6.54E-149	-0.3897	0.3810	0.5900	1.10E-144
FASN	6.51E-139	-0.3523	0.5840	0.7990	1.10E-134
SFRP1	1.38E-132	-0.6644	0.1660	0.3310	2.33E-128
FADS2	3.09E-104	-0.2767	0.3460	0.5290	5.20E-100
DGAT1	7.60E-93	-0.2873	0.4710	0.6230	1.28E-88
AGPAT2	3.80E-92	0.2510	0.7250	0.6250	6.40E-88
LPCAT1	7.14E-90	-0.2720	0.5560	0.7170	1.20E-85
CD3E	2.57E-60	-0.3002	0.1530	0.2540	4.32E-56
